# The contribution of dietary and plasma folate and cobalamin to levels of angiopoietin-1, angiopoietin-2 and Tie-2 receptors depend on vascular endothelial growth factor status of primary breast cancer patients

**DOI:** 10.1038/s41598-019-51050-x

**Published:** 2019-10-16

**Authors:** Saeed Pirouzpanah, Parisa Varshosaz, Ashraf Fakhrjou, Vahid Montazeri

**Affiliations:** 1 Molecular Medicine Research Center, Biomedicine Institute, Tabriz University of Medical Sciences, Tabriz, 5166614711 Iran; 2 Drug Applied Research Center/ and also Department of Biochemistry and Dietetics, Faculty of Nutrition and Food Sciences, Tabriz University of Medical Sciences, Tabriz, 5166614711 Iran; 3 Student’s Research Committee, Tabriz University of Medical Sciences, Tabriz, 5165665811 Iran; 4 Department of Pathology, School of Medicine, Tabriz University of Medical Sciences, Tabriz, 5156913193 Iran; 5 Department of Thoracic Surgery, Faculty of Medicine, Surgery Ward, Tabriz University of Medical Sciences, and also Nour-Nejat Hospital, Tabriz, 5138665793 Iran

**Keywords:** Breast cancer, Cancer epidemiology, Tumour angiogenesis

## Abstract

The aim of this study was to determine the association of dietary folate and cobalamin with plasma levels of Angiopoietins (ANG), vascular endothelial growth factor-C (VEGF-C) and tyrosine kinase receptor-2 (Tie-2) of primary breast cancer patients. Women (n = 177), aged 30 to 75 years diagnosed with breast cancer were recruited from an ongoing case series study. Dietary intake of nutrients was estimated by using a validated food frequency questionnaire. Enzyme-linked immunosorbent assay was applied to measure biomarkers. MCF-7 cell cultures were supplemented with folic acid (0–40 μM) for 24 h to measure cell viability and fold change of expression by the real-time reverse transcriptase-polymerase chain reaction. Structural equation modeling was applied to analyze the structural relationships between the measured variables of nutrients and Angiopoietins. Dietary intake of folate and cobalamin showed a significant inverse correlation with plasma ANG-1 and ANG-2 (P < 0.05), particularly in subjects with estrogen-receptor positive tumors or low plasma VEGF-C. Plasma folate was positively associated with the ratio of ANG-1/ANG-2 (P < 0.05). Residual intake levels of total cobalamin were inversely associated with plasma ANG-1 when plasma stratum of VEGF-C was high (P < 0.05). Structural equation modeling identified a significant inverse contribution of folate profiles on the latent variable of Angiopoietins (coefficient β = −0.99, P < 0.05). Folic acid treatment resulted in dose-dependent down-regulations on *ANGPT1* and *ANGPT1*/*ANGPT2* ratio but *VEGF* and *ANGPT2*/*VEGF* were upregulated at folic acid >20 μM. Studying the contributing role of dietary folate to pro-angiogenic biomarkers in breast cancer patients can infer the preventive role of folate in the ANGs/VEGF-C-dependent cascade of tumor metastasis. By contrast, high concentrations of folic acid *in vitro* supported *VEGF-C*-dependent *ANGPT2* overexpression might potentiate micro-lymphatic vessel development to support malignant cell dissemination.

## Introduction

Pathological angiogenesis is an irregular rapid proliferation of endothelial cells (EC) of blood vessels and *de novo* formation of new vessels from pre-existing vascular^1^. Angiogenesis, an important and complex process, is a rate-limiting determinant to the growth of tumoral neoplasms^2^. Indeed, pathologic angiogenesis entails capillaries outgrowth from the primary blood vessels (hemoangiogenesis) and lymphatic vessels (lymphangiogenesis)^3^. Lymphangiogenesis, the expansion of lymphatic system initiates the breast cancer invasion, and predispose metastasis to the regional lymphatic nodes^4^. Dissemination of tumor cells to regional lymphatic system facilitated greatly when intra-tumoral neo-lymphangiogenesis has been already displayed^5^. The formation of lymphatic macro-metastasis is a pathologic feature prognoses poor outcomes^6^, including metastatic involvement of axillary lymph nodes which accordingly associate with lung metastasis^5^.

Angiogenesis is the pivotal step in cancer propagation,^7^ induced by perturbations in the ratio of angiogenic stimulus in favor of promoting the proliferation and altered stability of vessel ECs^8^. Pro-angiogenic hypoxia-induced growth factors such as angiopoietin-2 (ANG-2) are responsible for pathologic angiogenesis in malignancies^1^. Once the over-regulation of vascular endothelial growth factor-C (VEGF-C) persists, it takes the lead in forming the lymphangiogenesis as a pathologic stage critical for metastasis of adenocarcinoma to other organs and spreading the malignancy^4^. Pathologic angiogenesis is distinct from physiologic angiogenesis which maintains the homeostasis of blood vessels in a quiescent state dependent on survival signals released from pericytes, such as VEGF-A and angiopoietin-1 (ANG-1)^9^.

VEGF isoforms are fundamental proliferative markers actively involved in the tumor growth, belong to the platelet-derived growth-factor/VEGF family^3^. Among those, VEGF-C is an isoform highly expressed in advance stages of malignant tumor invasion^10^. VEGF-C exerts its function by binding to a specific endothelial tyrosine kinase receptor, VEGFR-3, which is expressed predominantly in lymphatic endothelium. Interestingly, VEGF-C expression is highly expressed and secreted by hypoxic malignant tumoral cells^11–13^. On the other hand, VEGF-A is a critical growth factor in inducing hemoangiogenic process^1,14,15^. Binding to VEGFRs by VEGF-C could be non-specific dependent on proteolytic processing of VEGF-C^16^. However, processed VEGF-C binds significantly with higher affinity to VEGFR-3 than VEGFR-2^16^. It has been addressed, specifically in the progression of breast cancer metastasis that those factors repressed VEGF-C-mediated signaling can reduce the risk of lymph node metastasis and hold the promising potential to further address improvements on cancer survival^15^.

Angiopoietins are endothelial-based pro-angiogenic growth factors that are reported to influence vascular remodeling and maturation^1^. ANG-1, predominantly expressed by malignant cells, pericytes, and smooth muscle cells, mediates survival signaling of ECs^17^. The stability of vascularization is enhanced by ANG-1 through increasing the interaction of ECs in the matrices of extra-cellular vicinity and preservation of vessel integrity^3^. Another angiopoietin, ANG-2, is expressed in the region where the vascular remodeling takes place (angiogenic tip cells) by activated ECs^18^. The ANG-2 can actively antagonizes ANG-1 signaling pathway^3^. Carmeliet and Jain have pronounced that ANG-1 can demonstrate mutual pro- and anti-angiogenic activities by stabilizing vascular maturation, and repressing tumor cell extravasation, respectively^14^. Therefore, ANG-1 is suggested as a non-specific target for anticancer therapy^14^. On the other hand, inhibiting ANG-2 can repress potently the angiogenesis^19^. More importantly, ANG-2 overexpression usually occurs by transformation of zipper-like junctions into a button like pattern mediates lymphangiogenesis initiation during tumor growth^20^. The activity of ANG-2 to develop angiogenesis is context-dependent^20^. Lymphatic ECs contain endothelium-specific transmembrane tyrosine kinase receptors, tyrosine kinase receptor-2 (Tie-2) binds ANG-2 and agonize Tie-2 activity in favor of lymphatic vessel growth^20^. In Ang-2-knockdown animal models, lymphatic vessels failed to grow and suggested the importance of ANG-2 in repressing the lymphatic development perhaps in cancer^21^. The lymphangiogenic activity of ANG-2-expressing tumor cells even become augmented when VEGF-C is over-expressed in cancer cells^20^. Foretinib is an anti-angiogenic drug can suppress lymphangiogenesis by inhibiting VEGFR-3 and ANG-2 intracellular signalings^22^. Therefore, the cross-talk between ANG-2 and VEGF-C activities in promoting pathologic lymphangiogenesis is an interesting issue which can accordingly make them important targets to regress intra- and peripheral-tumoral lymphatic infiltrations^14^.

Different isoforms of Angiopoietins act through Tie-1 and Tie-2^1^. Tie-2 is the main receptor for Angiopoietins, ANG-1, and ANG-2, and mediates downstream signaling of angiogenesis^23^. Studies showed that the total plasma levels of Tie-2 also contained a measurement of soluble-Tie-2 (sTie-2)^24^. The breakdown of the external domain of Tie-2 through proteolytic cleavage could generate sTie2, and subsequently released into the circulation^24^. Likewise transmembrane Tie2, angiogenic growth factors can bind sTie2 but inhibit cellular ANG-1/Tie-2 signaling pathway, and consequently suppress new vascular formation^25^.

Numerous data showed the effects of dietary components on various aspects of cancer prevention and protection^26^, but investigations on pro-angiogenic effects of dietary factors on cancer progress are scarce and limited to experimental *in vitro* studies^27^. In this regard, an *in vitro* study has provided evidence of probable effects of folate, a one-carbon related nutrient, on up-regulation of ECs proliferation^28^. Folate and other one-carbon related nutrients are essential for a variety of biological processes. Folate deficiency and folate antagonists can disturb significantly cell proliferation (DNA synthesis), genetic integrity (e.g., uracil misincorporation), and aberrant epigenetic pro-carcinogenic reprogramming (DNA methylation, transcriptomic alterations, and chromosomal instability)^29^. The mRNA transcriptional levels seem far more crucial variable influenced by folate levels than the protein translation episodes and post-translation processes affected less by folate^30^. Evidence indicated that dietary intakes of folate and cobalamin have correlations with breast cancer risk^31^. In this context, only Lin *et al*.^28^ investigated the effects of folic acid treatment on cell proliferation of HUVEC cells suggesting dose-dependent inhibitory effects of folate on cell cycle arrest in G0/G1 phase potentially preventing new vascularizations^28^. Cobalamin is a nutrient exerting its metabolic functions as a co-enzyme intervening in methyl group transfer mediated by folate^31^. Generally, there is little information about the contributing role of folate and cobalamin on angiogenic biomarkers derived mainly from breast cancer cells.

The simultaneous inhibition of ANGs and VEGFs might be potently important as anti-angiogenic therapy and ameliorating lymphangiogenesis, and consequently control cancer progression^27^. However, further studies are necessary to determine the effects of folate and cobalamin on pro-angiogenic growth factors^28^. Therefore, we aimed to investigate the association of dietary and plasma levels of one-carbon nutrients, folate, and cobalamin, with plasma levels of Angiopoietins, VEGF-C and Tie-2 receptor in Iranian women with advanced breast cancer.

## Materials and Methods

### Study population

The participants were recruited from an ongoing consecutive case-series study, and details of the inclusion criteria are described in previous publications^32–34^. The current study recruited 177 women newly diagnosed with breast malignancy post partial or radical mastectomy and histopathological confirmation of infiltrative ductal carcinoma. Eligible women were recruited between 2013 to 2015 at the surgical ward of Nour-Nejat hospital, Tabriz, Iran. Patients were considered eligible if they did not have a medical history of any kind of neoplasm (benign or other malignancy in another anatomic site) prior to the recent diagnosis and had not been exposed to adjuvant or neoadjuvant therapy (chemotherapy, radiotherapy, targeted drug trastuzumab, and hormonal therapy). Other important eligibility criteria included breast cancer stage 3 (locally advanced BC) and stage 4 (metastatic BC) based on TNM staging method, no former or recent occurrence of acute or chronic disease (such as severe liver or kidney failure, hyperthyroidism, polycystic ovary syndrome and gastrointestinal inflammatory disorders), taking no longitude medications of methotrexate, sulfasalazine, anticonvulsants and anticontraceptive for two years before the study and not following folate or cobalamin-rich dietary patterns during the year prior to participating in this study^32,35^. Morbidly obese patients (BMI >40 kg/m2) were excluded. Face-to-face interviews were conducted by experienced interviewers. The sample size of this study was calculated using the mean difference formula (by considering α = 5% and β = 20%) and based on collected data of previous study^36^.

### Ethics approval

The study and ethical points described verbally to each participant and obtain written consent form prior to enrollment. Design and the procedure of conducting the study were carried out in accordance with the Ethical Guidelines for Observational Studies^37^. The research protocol outlining methodology, study subjects, sample size, data collection, biochemical tests and analysis, and related ethical considerations have been reviewed and received ethical approval by the Ethical Committee of Tabriz University of Medical Sciences (ethical code: 5-4-8327). This report was prepared according to the STROBE statements specified for observational studies^38^.

### Data collection

Demographic and general data were collected and have been reported previously^33^. Anthropometric measurements were performed at the time of interview for the current study. Information on whether participants had undergone bilateral oophorectomy and at what age was taken into account with regard to determining the age at menopause.

Pathological data for each participant, including histopathological subtypes of invasive carcinoma (ductal and non-ductal), tumor size and grade and immunohistochemistry staining-based expression statuses of estrogen receptor (ER), progesterone receptor (PR) and human epidermal growth factor receptor 2 (EGFR-2 or HER2/neu) was obtained by reviewing pathology results of each patient. In general, tumor samples were fixed in 10% buffered formalin and embedded as paraffin blocks. Immunohistochemical (IHC) staining was performed by applying primary antibodies on deparaffinized tumors sections and incubated them for an overnight at 8 °C. The percentage of cells stained for antibodies was verified by a binocular microscope (Zeiss KF2 binocular, Germany). The positivity of HER2 was defined when the membrane/membrane plus cytoplasmic staining by antibody (A0485, 1/200; Dako Denmark A/S) classified as the weak or greater intensity at ≥10% of tumor cells^39^. The antibody used for ER staining was Clone ID5; Dako Denmark A/S (Glostrup, Denmark). The PR staining was carried out using Clone PgR636; Dako Denmark A/S (Glostrup, Denmark). The tumoral status for epxression of ER and PR proteins were considered positive when tumor cells had been stained more than 5%^40^. The invasive breast cancer was classified to five subgroups based on IHC data provided by Parise *et al*. as follow: luminal A (ER+, PR+, HER2-, grade: I/II), luminal B/HER2+ (ER+, PR+/−, HER2+, grade: III), luminal B/HER-(ER+, PR+/−, HER2-, grade: III), HER2-enriched (ER-, PR-, HER2+) and triple-negative (ER−, PR−, HER2−)^41^.

### Dietary assessment

Dietary information about the intake levels of B-group vitamins was obtained by means of a validated food frequency questionnaire (FFQ) containing 136 food items within 10 main food groups^29,35,42^. Nutritionist IV software (version 3.5.2; 1994, N-Squared Computing, San Bruno, CA) was used to establish daily intake of nutrients consumed by each patient. Total dietary intake of nutrients is the sum of daily dietary intake from food and a daily estimates of supplements consumed. The residual method was used to estimate the amount of nutrient independent of the effect of total calorie intake (energy-adjusted amount of nutrient) and calculated for each person as described by Willett^43^.

The model accuracy to show the association between dietary nutrients (folate and cobalamine) and plasma biomarkers (total homocysteine, folate, and cobalamin) had been met and documented previously^29,35^. However, at present study, again the validity of the FFQ to assess the intake levels of folate and vitamin B_12_ was evaluated in accordance with plasma levels of folate and B_12_ through stratification analyses (Supplementary Table 1).

### Plasma biochemical assays

Venous blood samples were taken following an overnight fast and prior to surgery. Plasma was extracted following centrifugation at 10000 rpm for 10 minutes at room temperature. The plasma was aliquoted and immediately stored at −80 °C freezer until required for testing.

Plasma levels of ANG-1 (Cusabio, Cat. No. CSB-EL001706HU), ANG-2 (Cusabio, Cat. No. CSB-E04500h), Tie-2 (Cusabio, Cat. no. CSB-EL023375HU), VEGF-C (eBioscience, Cat. No. BMS297/2), folate (Monobind, Cat. No. 7525-300) and cobalamin (Monobind, Cat. No. 7526-300) were measured by enzyme-linked immunosorbent assay (ELISA) using commercially available kits according to the manufacturer’s instructions. The coefficients of variation for within and between assays of all biochemical measures were estimated to be <10%. The measurement of each biomarker was performed at the same time in one laboratory run.

### Cell culture

Folic acid calcium salt was purchased from Dana Pharma Co., Tabriz (Iran), which they had purchased it from DSM Nutritional Products Ltd. (Basel, Switzerland). Folic acid was dissolved in water using sodium hydroxide solution (1 M, 50 mg/ml) to prepare dilution at 1 mg/ml (1.96 mM).

Human breast adenocarcinoma cell line (MCF-7) was purchased from the Pasteur Institute, National Cell Bank of Iran (Iran). Primarily, MCF-7 was grown in RPMI-1640 medium (GIBCO-Thermo Fisher Scientific, USA), 10% fetal bovine serum (Sigma-Aldrich, St. Louis, MO), penicillin (100 U/ml), streptomycin (100 μg/ml), and amphotericin B (0.25 μg/ml). Cells were incubated in nonsynchronous cultures in a humidified atmosphere at 37 °C with 5% CO_2_. Folic acid concentrations were prepared at 5, 10, 20 and 40 μM in the mode of independent triplicate experiments. Media with no addition of folic acid was considered as control (triplicate). Selected folic acid concentrations were chosen within the range of 0–200 μM of folic acid based on the proportion of cell viability results. The number of viable cells (with active metabolism) present in media was evaluated by MTT assay using 3-(4,5-dimetylthiazol-2-yl)−2,5-diphenyltetrazolium bromide) (Roche Diagnostics GmbH, Mannheim, Germany). The rate of cellular proliferation in cultures was tested by adding 10 µg of 5 mg/ml MTT to each well in 96-well plates. The average confluence of cells was 7000 within each well. Each well contained a mixture of culture media (200 μl) for 24 h at 37 °C with 5% CO_2_ and then solubilized with dimethyl sulfoxide (DMSO). Sorenson buffer (25 μl) was also added to the media. The quantification of formazan was measured at 540 nm using an ELISA plate reader (BioTeck, Bad Friedrichshall, Germany), which is inversely associated with the number of viable cells at a higher intensity of purple color. All outputs were then analyzed and normalized relative to the untreated cells. The number of cells was counted using an inverted microscope to record the number of viable or nonviable cells from three independent experiments. The clonogenic assay or colony formation assay was used to investigate the growth ability of MCF-7 in treated media with 20 μM of folic acid. The MCF-7 cells were allowed to grow in 6 well-plates at 37 °C for 24 h till 120 h to reach an average confluence of almost 2000 cells/well. Methanol-acetic acid (3:1 ratio) was used to fix grown colonies and then stained with Giemsa solution (10%).

### RNA extraction and real-time reverse transcriptase-polymerase chain reaction (PCR)

Total cellular RNA was extracted from cells using RNX-Plus reagent (Cinagen Co., Tehran, Iran) according to the manufacturer’s instruction.

The concentration of the purified RNA was determined by measuring absorbance at 260 and 280 nm. The quantity of messenger RNA (mRNA) was measured by means of Nanodrop ND-1000 (Nanodrop Technologies, DE, USA). Total mRNA was first normalized in concentration (up to 2 μg/ml) and applied to synthesize complementary DNA (cDNA) using HyperScript Reverse Transcriptase following the manufacturer’s protocol (HyperScript First-Strand Synthesis Kit, GeneAll, South Korea). The reaction mixture for real-time PCR was prepared by combining 12.5 μl RealQ Plus 2x Master Mix Green (AMPLICON, Denmark), 1 μl of each primer (10 μM), PCR-grade distilled water and template cDNA (average 100 ng/μl) in a total volume 25 μl. The PCR primer sequence for genes was listed in Supplementary Table 2. The annealing temperature for reactions was 62 °C. Each sample was amplified in triplicate experiments. The relative expression was quantified using 2^−Δct (cycle threshold)^ formula using *HGPRT* (*hypoxanthine-guanine phosphoribosyltransferase*) transcription levels as a reference gene^44^. Fold change of expression was also measured using 2^−ΔΔct^ formula^44^.

### Statistical analysis

Linear regression analysis was used to detect the correlation coefficient (β) between intake levels of nutrients and plasma levels of biomarkers. Logarithmic (log) transformation was performed for all continuous variables except for Tie-2 (plasma variable), folate (plasma and dietary variables) and cobalamin (plasma variable). Log transformation was conducted to attenuate variations relevant to errors and verify the normality of the data distribution within each sub-category of a variable. Odds ratio (OR) and 95% confidence interval (95% CI) was calculated to analyze the association of dietary and total intake levels of a nutrient categorized based on the median of a variable in the study population and dietary reference intake (DRI). Partial correlation coefficient values (r) were also performed between dietary intake of nutrients and plasma level of biomarkers in both crude and adjusted models (controlled for confounding factors). The main confounding factors included age at diagnosis (y), body mass index (BMI; kg/m^2^), the frequency of live-birth delivery (n) and histopathological grade of disease (І, ІІ, ІІІ). Mean values of expression levels at each concentration of folic acid were compared using analysis of variance (ANOVA) followed by Dunnett test. Three samples for each experiment with certain folic acid concentration were analyzed. The effective concentration of folic acid induces a 50% growth response (half-maximal response) in the treatment of MCF-7 after 24 h was determined as EC_50_. The EC_50_ curve was plotted by considering cell viability percentage in verticle Y-axis and logarithm transformed of folic acid concentration on the horizontal X-axis.

Structural equation modeling (SEM) was performed using AMOS (IBM SPSS AMOS ver. 16) to test the goodness-of-fit (adequacy) of the conceptual theoretical model based on the hypothesis of the present study, that is, to reveal the contribution of folate and cobalamin to plasma levels of Angiopoietins. Firstly, dietary variables highly correlated with Angiopoietins in univariate and multivariate analyses were screened and included in the measurement model of SEM. Independent latent dietary variables (exogenous variable) were constructed to summarize the intercorrelation of indicator variables measured both in plasma and FFQ. The path diagram was specified to show the simplest form of the theoretical regression model. Thereafter, a multiple regression model was identified after displaying potent covariates. In general, maximum likelihood estimation (MLE) was performed to estimate the model parameters. Model estimation resulted in measuring the standardized regression weight as beta (β) weights used to show standardized partial model coefficients. The goodness-of-fit of the model was evaluated according to criteria cited by Bentler and Marsh^45,46^. Primarily, the root means a square error of approximation (RMSEA) used as a global fit measure, when becomes below 0.05 indicates an acceptable model fit. The normed chi-square, that is the value of chi-square divided by a given degree of freedom (CMIN/df) is a criterion to show close fit when the value is <5. Several additional criteria used as global indices to test the model goodness-of-fit. Comparative fit index (CFI), incremental fit index (IFI), normed fit index (NFI) and Tucker-Lewis index (TLI) were used to verify how the model fits the best the data. For these measures, the values close to 0.95 reflect the good fit of the model (1.00 implies a perfect fit).

Data analyses were carried out using the SPSS statistical package for Windows (version 17; SPSS Inc, Chicago, IL, USA). Two-tailed *P* < 0.05 was considered the level of significance.

### Ethical approval and consent to participate

All study participants provided informed consent form and study protocol was approved by the Ethical Committee of Tabriz University of Medical Sciences (ethical code: 5-4-8327).

## Results

### Study participants

The number of eligible study participants was 177. The mean age at diagnosis of our sample population was 46.0 ± 8.5 years in an age range of 30 to 80 years. The relative frequency of postmenopausal women with BC was 68.8% (117 out of 170), and premenopausal women comprised 31.2% (53 out of 170) of the study population (P < 0.05). The frequency of histopathologic data, dietary and plasma status of nutrients (folate and cobalamin), gynecological characteristics of the study population are described in Table 1. The pathological grade ІІ was as high as 74.8% (116 out of 145) and significantly different from other grades (P < 0.001). Invasive ductal carcinoma (IDC) was the predominant histopathological subtypes of breast cancer at 73.8% of the studied population (P < 0.01). The molecular subtypes of breast cancer of the study population (n = 128, there was some missing data) included luminal A 71.1% (n = 91 out of 128), luminal B/HER2-positive 15.6% (n = 20), luminal B/HER2-negative 6.3% (n = 8), HER2-enriched 4.7% (n = 6), and triple-negative 2.3% (n = 3).

**Table 1:** Histopathology and general characteristics of participants with breast cancer (N = 177).

Variable	Total patients (n)	The relative frequency (%)	P-value*
**Age at diagnosis**			
Mean ± S.D.	172	46.0 ± 8.6	
<46	84	48.8	
≥46	88	51.1	0.09
**Histopathology**			
Ductal	130	73.8	
Others	15	8.5	<0.01
**Histopathologic grade**			
I	17	11.7	
II	116	80.0	
III	12	8.2	<0.01
**Tumor size**			
<1.99	27	17.5	
2.0–4.99	102	66.2	
≥5	25	16.2	<0.01
**Menopausal status**			
Premenopause	117	68.8	
Postmenopause	53	31.2	<0.01
**Number of live birth**			
<2	28	16.5	
≥2	141	83.4	<0.01
**Number of lactation**			
<2	32	19.3	
≥2	133	80.6	<0.01
**BMI (kg/m2)**			
<24.99	26	16.6	
25–29.99	77	49.0	
≥30	54	34.4	<0.01
**Dietary folate (µg/d)** ^**a**^			
<400	107	61.1	
≥400	68	38.9	<0.01
**Dietary cobalamin (µg/d)**			
<2.4	35	20.1	
≥2.4	139	79.9	<0.01
**Plasma folate (ng/ml)** ^**b**^			
<4.5	12	6.8	
≥4.5	165	93.2	<0.01
**Plasma cobalamin (pg/ml)**			
<200	39	22.2	
≥200	136	77.8	<0.01

Note: Some missing data including age at diagnosis (n = 8), histopathological data (n = 35), histopathological grade (n = 35),tumor size (n = 26), menopausal status (n = 10), number of live birth (n = 11) number of lactation (n = 15), BMI (n = 23). *The *p*-value obtained by performing chi-square test.

^a^Dietary and total folate and cobalamin intakes were classified based on dietary reference intake (DRI)^35^. ^b^Plasma concentration of folate 2–20 ng/ml and cobalamin 200–900 pg/ml were used as reference values according to the protocol of kits (folate: Cat N. 7525-300 and cobalamin: Cat N. 7526-300).

### Dietary assessment analyses

**Figure 1:**
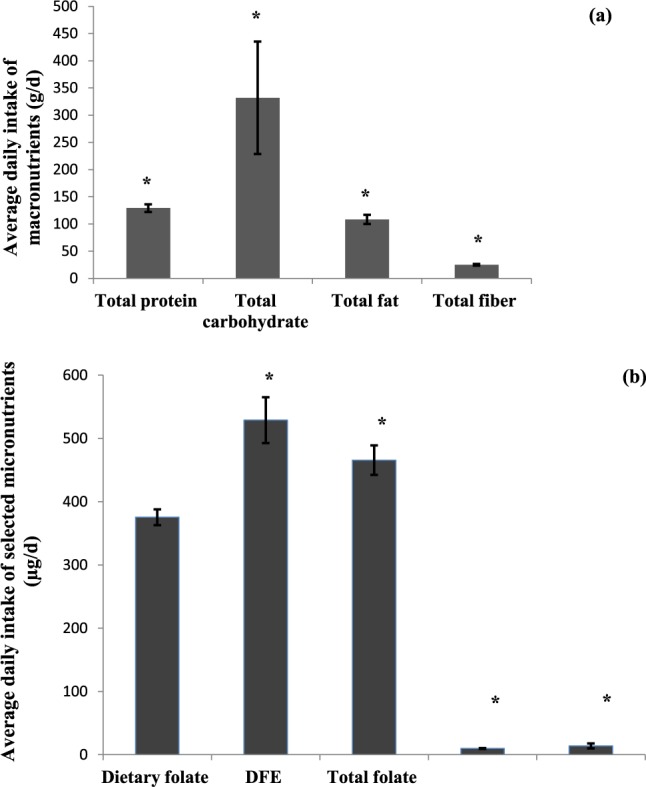
Dietary intakes of patients. Daily dietary intake of macronutrients including total intake levels of protein, carbohydrate, fat and fiber (graph A), dietary and total (dietary plus supplemented amounts) intake levels of folate and cobalamin and dietary folate equivalent (DFE: μg/d of food folate plus 1.7 times the μg/d of supplemented folic acid) (graph B) were compared with their dietary reference intake (DRI). Age- and gender-dependent reference amounts of intake based on DRI^26^ were as follow: carbohydrate ≥130 g/d, protein ≥46 g/d, fat ≥27.5 g/d (based on DRI 2002/2005), fiber ≥23 g/d, folate ≥400 µg/d and cobalamin ≥2.4 µg/d (based on DRI 1998). *p < *0.05*. Mean values ± S.D. are presented. µg/d, microgram/day; ng/d, nanogram/day.

Average daily intake of total calories, macronutrients and fiber are presented in Fig. 1a and compared with dietary reference intake (DRI). Average intake of total calorie (3003 ± 116 kcal/d versus DRI: 2500 kcal/d), carbohydrate (332 ± 103 g/d versus DRI: 130 g/d), fat (108 ± 8 g/d versus DRI: 27.5 g/d), and protein (129 ± 7.0 g/d versus 46.0 g/d) were greater than the DRI required for a healthy population in age- and gender-dependent manner (P < 0.01). The mean daily amount of folate and cobalamin were 375 ± 12 µg/d, and 6.1 ± 0.6 µg/d, respectively (Fig. 1b). Total amounts of folate and cobalamin consumed were 465 ± 23 and 14.0 ± 3.9 µg/d, respectively (Fig. 1b). The median dietary folate intake of study subjects was 352 µg/d (5th-95th percentile: 150–668) and intake level of cobalamin was 3.7 µg/d (5th-95th percentile: 1.0–18.7). The DRI value of folate is 400 µg/d^26^. It was noted that the average intake levels of cobalamin among our population was estimated at about four-fold higher than the reference value determined for cobalamin (DRI of cobalamin is 2.4 µg/d)^26^.

### Plasma biochemical assays

**Figure 2:**
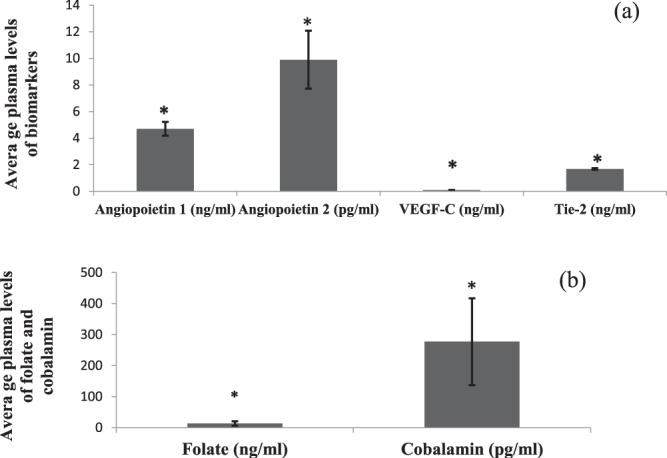
Plasma levels of angiogenic markers, folate and cobalamin were compared with the upper limit of the normal range measured in healthy individuals (**a**,**b**). Normal ranges were ascribed as follows: ANG1 0.600–6.000 ng/ml, ANG2 0.500–3.000 ng/ml, Tie2 10–92 ng/ml, VEGF-C 0.0–0.50 ng/ml, folate 2–20 ng/ml and cobalamin 200–900 pg/ml according to protocol of kits (folate: Cat N. 7525-300 and cobalamin: Cat N. 7526-300)^47,52^. *p < *0.05*. Mean values ± S.D. are presented.

The mean plasma concentration of ANG-1 was 4.7 ± 7.0 ng/ml, ANG-2 was 9.9 ± 28.7 pg/ml, Tie-2 was 1.6 ± 0.8 ng/ml, and VEGF-C was 0.10 ± 0.18 ng/ml in the study population (Fig. 2a). Plasma concentrations of folate (13.3 ± 7.1 ng/ml), and cobalamin (277 ± 140 pg/ml) were also significantly higher than the upper limit rates characterized as the normal range in healthy normotensive individuals by other researches using ELISA^47^ (P < 0.001) (Fig. 2b).

### Validity tests of dietary data of folate and cobalamin

The supplementary analyses were performed to detect the diagnostic accuracy of dietary and total folate and cobalamin with respect to the measurements of their plasma levels and summarized in Supplementary Table 1. In this context, the area under the curve (AUC) for dietary folate evaluated by FFQ was significantly correlated with plasma levels in the reference model at AUC = 0.67 [95% CI: 0.53–0.81; P < 0.01, cutoff point (COP): 280 µg/d]. Data of total folate intake obtained from FFQs demonstrated a good performance to detect low folate plasma levels with AUC = 0.71 (95% CI: 0.58–0.84; P < 0.01, COP: 374 µg/d). However, variables showing the intake levels of cobalamin from FFQs were not correlated significantly with plasma levels of cobalamin (Supplementary Table 1).

### Univariate and multivariate regression modeling

**Figure 3:**
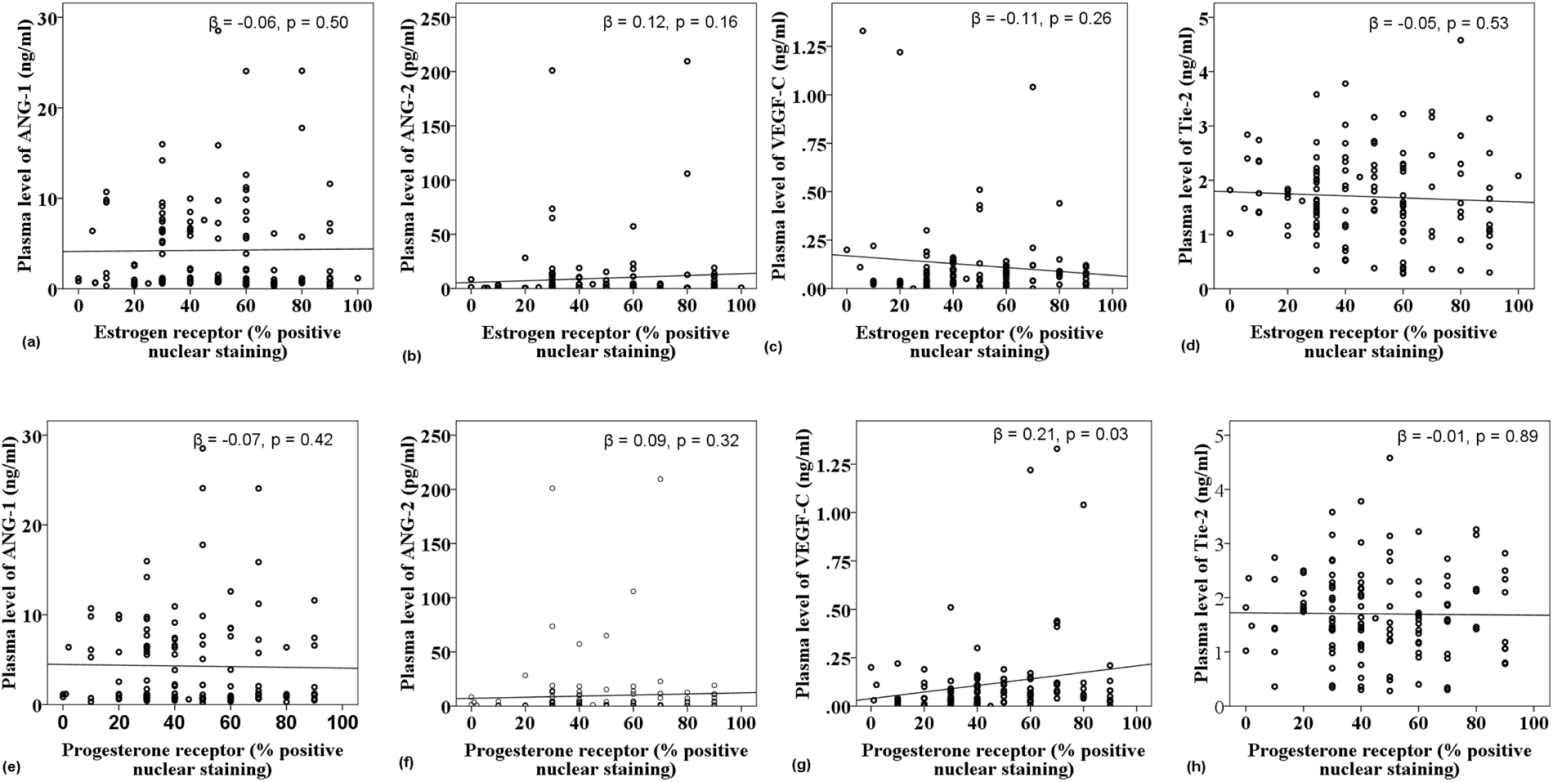
Scatter plots depicted to show the crude correlation of protein expression levels of ER (**a**–**d**) and PR (e-h) with plasma levels of studied angiogenic growth factors in primary breast cancer patients. The linear regression analysis has been done to obtain standardized beta (β). *p* < *0.05* considered statistically significant.

Linear regression analysis was performed between dietary and plasma levels of nutrients and plasma levels of Angiopoietins and their ratio in the crude model (unadjusted) and multivariate model 1 (adjusted for age at diagnosis and BMI) and model 2 (adjusted for frequency of live-birth delivery and grade of disease in addition to age at diagnosis and BMI) (Table 2). Dietary intake levels of folate (β = −0.20, P < 0.05) and cobalamin (β = −0.17, P < 0.05) showed significant negative associations with plasma levels of ANG-2 in model 1. Total folate intake was also correlated inversely with ANG-2 in model 1 (β = −0.19, P < 0.05). Residual total cobalamin intake showed an inverse relationship with the ratio of ANG-1/ANG-2 (β = −0.18, P < 0.05). A positive correlation between plasma folate level and ANG-1/ANG-2 was obtained after adjusting for the related confounders (β = 0.25, P < 0.05). Moreover, Fig. 3 shows linear regression analyses between plasma levels of ANGs, Tie-2, and VEGF-C and tumoral expression levels of hormone receptors (IHC data). The high proportion of tumor cells with PR-positivity was significantly associated with high plasma levels of VEGF-C (β = 0.21, P < 0.05).

**Table 2:** Linear regression analysis and correlation coefficients (β) between variables of dietary, total and plasma level of nutrients with the plasma level of studied biomarkers and the corresponding ratio in the unadjusted and adjusted model (N = 177).

Variable	ANG1	ANG2	Tie-2	ANG1/ANG2	ANG1 + ANG2/Tie-2	ANG2/Tie-2
**Nutrient intake level (µg/d)**						
**Dietary folate**						
Crude	0.13 (0.09)^a^	−0.13 (0.08)^a^	−0.04 (0.57)^a^	0.02 (0.74)^a^	−0.06 (0.41)^a^	−0.06 (0.44)^a^
Model1^b^	−0.09 (0.23)	**−0.20 (0.01)**	0.01 (0.98)	0.09 (0.24)	−0.12 (0.12)	−0.14 (0.07)
Model2^c^	−0.07 (0.41)	−0.18 (0.05)	−0.01 (0.98)	0.10 (0.25)	−0.11 (0.23)	−0.14 (0.12)
**Total folate**						
Crude	−0.12 (0.10)	**−0.15 (0.04)**	−0.01 (0.92)	0.01 (0.99)	−0.01 (0.87)	−0.01 (0.84)
Model1	−0.08 (0.28)	**−0.19 (0.01)**	0.02 (0.72)	0.02 (0.78)	−0.04 (0.57)	−0.06 (0.41)
Model2	−0.12 (0.18)	−0.13 (0.17)	−0.02 (0.80)	0.06 (0.52)	0.02 (0.76)	0.02 (0.79)
**DFE**						
Crude	−0.12 (0.10)	−0.09 (0.20)	−0.02 (0.76)	−0.02 (0.73)	0.05 (0.44)	0.04 (0.54)
Model1	−0.13 (0.09)	−0.12 (0.14)	−0.01 (0.87)	−0.04 (0.61)	0.05 (0.51)	0.02 (0.74)
Model2	−0.09 (0.31)	−0.06 (0.52)	−0.04 (0.65)	−0.02 (0.79)	0.10 (0.27)	0.09 (0.29)
**Dietary cobalamin**						
Crude	0.01 (0.99)	**−0.15 (0.04)**	−0.14 (0.06)	−0.02 (0.73)	0.03 (0.65)	−0.03 (0.61)
Model1	−0.02 (0.80)	**−0.17 (0.03)**	−0.14 (0.07)	−0.04 (0.61)	−0.01 (0.90)	−0.06 (0.42)
Model2	0.01 (0.83)	−0.11 (0.20)	−0.16 (0.07)	−0.01 (0.99)	0.09 (0.31)	0.02 (0.82)
**Total cobalamin**						
Crude	−0.06 (0.41)	−0.14 (0.05)	−0.01 (0.81)	−0.08 (0.28)	−0.01 (0.85)	−0.04 (0.53)
Model1	−0.07 (0.33)	−0.15 (0.06)	−0.01 (0.87)	−0.11 (0.15)	−0.04 (0.61)	−0.05 (0.49)
Model2	−0.07 (0.40)	−0.08 (0.38)	−0.01 (0.84)	−0.09 (0.32)	0.04 (0.66)	0.03 (0.73)
**Residual intake (µg/d)**						
**Dietary folate**						
Crude	**−0.18 (0.01)**	−0.14 (0.06)	0.01 (0.90)	0.01 (0.91)	0.11 (0.14)	−0.07 (0.30)
Model1	**−0.18 (0.02)**	**−0.22 (0.01)**	0.07 (0.34)	0.08 (0.33)	**−0.20 (0.01)**	**−0.18 (0.02)**
Model2	−0.17 (0.05)	**−0.19 (0.03)**	0.08 (0.35)	0.09 (0.31)	**−0.20 (0.02)**	−0.17 (0.05)
**Total folate**						
Crude	−0.12 (0.12)	−0.11 (0.12)	−0.01 (0.83)	−0.02 (0.76)	0.02 (0.72)	0.02 (0.75)
Model1	−0.09 (0.25)	−0.15 (0.05)	0.01 (0.91)	−0.02 (0.79)	0.01 (0.96)	−0.01 (0.85)
Model2	−0.13 (0.17)	−0.09 (0.31)	−0.01 (0.85)	−0.01 (0.96)	0.05 (0.58)	0.05 (0.56)
**Dietary cobalamin**						
Crude	−0.03 (0.66)	−0.12 (0.09)	−0.11 (0.14)	−0.01 (0.83)	0.08 (0.25)	0.02 (0.73)
Model1	−0.07 (0.37)	−0.15 (0.05)	−0.08 (0.27)	−0.03 (0.68)	0.01 (0.87)	−0.02 (0.75)
Model2	−0.05 (0.58)	−0.10 (0.24)	−0.09 (0.31)	−0.01 (0.87)	0.09 (0.29)	0.05 (0.55)
**Total cobalamin**						
Crude	−0.07 (0.32)	−0.06 (0.39)	0.09 (0.22)	−0.12 (0.12)	−0.01 (0.97)	−0.01 (0.90)
Model1	−0.10 (0.19)	−0.05 (0.48)	0.08 (0.29)	−0.15 (0.06)	−0.01 (0.87)	−0.01 (0.97)
Model2	−0.12 (0.19)	−0.01 (0.85)	0.09 (0.32)	**−0.18 (0.04)**	0.02 (0.80)	0.04 (0.59)
**Plasma level**						
**Folate (ng/ml)**						
Crude	−0.13 (0.07)	−0.01 (0.92)	−0.03 (0.61)	**0.15 (0.04)**	−0.11 (0.13)	−0.07 (0.34)
Model1	−0.13 (0.09)	−0.01 (0.85)	−0.02 (0.76)	**0.19 (0.02)**	−0.10 (0.18)	−0.06 (0.43)
Model2	−0.17 (0.05)	0.04 (0.67)	0.04 (0.61)	**0.25 (0.01)**	−0.10 (0.23)	−0.03 (0.67)
**Cobalamin (pg/ml)**						
Crude	0.11 (0.14)	0.01 (0.85)	−0.08 (0.24)	−0.06 (0.42)	0.01 (0.98)	−0.04 (0.55)
Model1	0.11 (0.18)	0.03 (0.69)	−0.10 (0.19)	−0.08 (0.34)	−0.01 (0.96)	−0.04 (0.56)
Model2	0.05 (0.54)	0.07 (0.45)	−0.09 (0.32)	−0.01 (0.84)	−0.05 (0.60)	−0.03 (0.67)

ANG1; angiopoietin 1, ANG2; angiopoietin 2, DFE; dietary folate equivalent.

^a^Data were expressed as β (*p-value*). The Statistically significant finding is shown in bold.

^b^The model 1 was adjusted for age at diagnosis (yr) and body mass index (BMI) at diagnosis (kg/m2).

^c^Adjusted for age at diagnosis (yr), body mass index (BMI) at diagnosis (kg/m2), frequency of live-birth delivery (n) and pathological grade of disease (І/ІІ/ІІІ).

### Hormonal receptor status

Stratification analysis to estimate the OR of association between dietary and total intake of folate and cobalamin with the plasma level of ANG-1 within subgroups of tumor hormone receptor status are shown in Table 3. Higher dietary folate intake was associated significantly with lower plasma levels of ANG-1 among HER2 positive breast cancer patients (OR = 0.18, 95% CI: 0.03–0.97). The residual total folate above the median level was associated with a lower plasma level of ANG-1 in subgroups of ER + (OR = 0.43, 95% CI: 0.21–0.87), and PR + patients (OR = 0.46, 95% CI: 0.22–0.94) (Table 3).

**Table 3:** Odds ratios and 95% confidence intervals (CIs) of dietary and total intakes and plasma levels of folate and cobalamin in association with the plasma level of angiopoietin 1 by considering receptor status (N = 177).

Plasma level of angiopoietin 1^a^						
Variable	ER		PR		HER2	
	Positive (n = 136)	Negetive (n = 13)	Positive (n = 131)	Negetive (n = 17)	Positive (n = 29)	Negetive (n = 120)
**Nutrient intake (µg/d)**						
**Dietary folate**						
<280^c^	1.00	1.00	1.00	1.00	1.00	1.00
≥280	0.54 (0.25–1.16)	0.33 (0.02–5.02)	0.59 (0.27–1.27)	0.20 (0.01–2.20)	**0.18 (0.03–0.97)**	0.68 (0.30–1.56)
<352^d^	1.00	1.00	1.00	1.00	1.00	1.00
≥352	0.63 (0.31–1.25)	0.75 (0.08–6.71)	0.68 (0.33–1.37)	0.50 (0.07–3.55)	0.37 (0.08–1.66)	0.73 (0.35–1.52)
<400^e^	1.00	1.00	1.00	1.00	1.00	1.00
≥400	0.64 (0.31–1.32)	1.33 (0.14–11.92)	0.66 (0.31–1.37)	0.80 (0.11–5.40)	0.60 (0.12–2.83)	0.77 (0.33–1.51)
**Total folate**						
<280^c^	1.00	1.00	1.00	1.00	1.00	1.00
≥280	0.56 (0.25–1.23)	0.33 (0.02–5.02)	0.61 (0.27–1.36)	0.20 (0.01–2.60)	0.80 (0.34–1.89)	**0.11 (0.01–0.71)**
<382^d^	1.00	1.00	1.00	1.00	1.00	1.00
≥382	**0.46 (0.22–0.92)**	0.75 (0.08–6.71)	0.49 (0.24–1.00)	0.50 (0.07–3.55)	0.55 (0.26–1.15)	0.26 (0.05–1.26)
<400^e^	1.00	1.00	1.00	1.00	1.00	1.00
≥400	0.55 (0.27–1.10)	1.33 (0.14–11.92)	0.59 (0.29–1.21)	0.80 (0.11–5.40)	0.67 (0.32–1.41)	0.35 (0.07–1.63)
**Dietary cobalamin**						
<2.4^e^	1.00	1.00	1.00	1.00	1.00	1.00
≥2.4	1.06 (0.47–2.38)	0.57 (0.30–1.08)	0.91 (0.39–2.09)	0.55 (0.31–0.99)	0.91 (0.15–5.53)	1.42 (0.60–3.37)
<3.7^d^	1.00	1.00	1.00	1.00	1.00	1.00
≥3.7	1.02 (0.51–2.04)	3.75 (0.27–51.37)	1.00 (0.49–2.01)	2.40 (0.30–19.40)	1.16 (0.26–5.05)	1.10 (0.53–2.30)
**Total cobalamin**						
<2.4^e^	1.00	1.00	1.00	1.00	1.00	1.00
≥2.4	1.07 (0.46–2.44)	0.57 (0.30–1.08)	0.91 (0.38–2.13)	0.55 (0.31–0.99)	0.56 (0.07–4.00)	1.59 (0.66–3.81)
<4.0^d^	1.00	1.00	1.00	1.00	1.00	1.00
≥4.0	0.75 (0.37–1.50)	12.50 (0.83–186.29)	0.72 (0.35–1.46)	6.00 (0.72–49.83)	0.66 (0.14–3.01)	1.03 (0.49–2.16)
**Residual nutrient (µg/d)**						
**Dietary folate**						
<374^f^	1.00	1.00	1.00	1.00	1.00	1.00
≥374	0.67 (0.33–1.33)	0.75 (0.08–6.71)	0.72 (0.36–1.46)	0.50 (0.07–3.55)	0.37 (0.08–1.66)	0.78 (0.37–1.63)
**Dietary cobalamin**						
<3.4^f^	1.00	1.00	1.00	1.00	1.00	1.00
≥3.4	0.55 (0.27–1.11)	6.66 (0.48–91.33)	0.49 (0.24–1.00)	6.00 (0.72–49.83)	0.88 (0.20–3.90)	1.10 (0.53–2.31)
**Total folate**						
<465^f^	1.00	1.00	1.00	1.00	1.00	1.00
≥465	**0.43 (0.21–0.87)**	0.75 (0.08–6.71)	**0.46 (0.22–0.94)**	0.50 (0.07–3.55)	0.26 (0.05–1.26)	0.51 (0.24–1.07)
**Total cobalamin**						
<3.8^f^	1.00	1.00	1.00	1.00	1.00	1.00
≥3.8	0.66 (0.33–1.33)	2.66 (0.27–25.63)	0.63 (0.31–1.29)	2.08 (0.29–14.54)	0.88 (0.20–1.90)	0.72 (0.34–1.51)

ER; estrogen receptor, PR; progesterone receptor, HER2; human epidermal growth factor receptor 2.

^a^The classification of angiopoietin 1 was based on the median plasma level of our studied population.

^b^All variables were categorized in the dichotomous group.

^c^Dietary and total intakes of folate were classified based on the cut of point obtained in our sample population.

^d^Dietary and total folate and cobalamin intakes were classified based on the median intake of studied BC participants.

^e^Dietary and total folate and cobalamin intakes were classified based on dietary reference intake (DRI).

^f^Energy-adjusted models (residual). Just median-based classification of residual dietary and total folate and cobalamin intakes of BC studied population considered in this model. Statistical significant finding is shown in bold (*p-*value < 0.05).

The estimated OR of dietary intake of folate and cobalamin in association with increased plasma levels of ANG-2 in various subtypes of hormonal receptors are also demonstrated in Table 4. The dietary folate intake was inversely associated with plasma levels of ANG-2 within BC sub-group characterized by ER+ (OR = 0.45, 95% CI: 0.22–0.92) and PR+ (OR = 0.42, 95% CI: 0.20–0.87). Intake levels of total cobalamin were associated inversely with the chance of detecting higher plasma levels of ANG-2 in ER-negative (OR = 0.33, 95% CI: 0.13–0.84) and PR-negative patients (OR = 0.33, 95% CI: 0.15–0.74).

**Table 4:** Odds ratios and 95% confidence intervals (CIs) of dietary and total intakes and plasma levels of folate and cobalamin in association with the plasma level of angiopoietin 2 by considering receptor status (N = 177).

Plasma levels of angiopoietin 2^a^						
Variable	ER		PR		HER2	
	Positive (n = 136)	Negetive (n = 13)	Positive (n = 131)	Negetive (n = 17)	Positive (n = 29)	Negetive (n = 120)
**Nutrient intake (µg/d)**						
**Dietary folate**						
<280^c^	1.00	1.00	1.00	1.00	1.00	1.00
≥280	0.69 (0.32–1.46)	0.12 (0.01–2.17)	0.64 (0.30–1.39)	0.30 (0.02–3.13)	0.46 (0.09–2.22)	0.58 (0.25–1.56)
<352^d^	1.00	1.00	1.00	1.00	1.00	1.00
≥352	**0.45 (0.22–0.92)**	0.80 (0.07–8.47)	**0.42 (0.20–0.87)**	1.07 (0.12–8.97)	0.31 (0.06–1.59)	0.50 (0.23–1.06)
<400^e^	1.00	1.00	1.00	1.00	1.00	1.00
≥400	0.66 (0.32–1.36)	1.25 (0.11–13.24)	0.68 (0.32–1.42)	1.50 (0.18–12.45)	0.73 (0.14–3.79)	0.65 (0.30–1.38)
**Total folate**						
<280^c^	1.00	1.00	1.00	1.00	1.00	1.00
≥280	0.71 (0.32–1.57)	0.12 (0.01–2.17)	0.67 (0.30–1.48)	0.30 (0.02–3.13)	0.35 (0.07–1.78)	0.66 (0.27–1.58)
<382^d^	1.00	1.00	1.00	1.00	1.00	1.00
≥382	0.97 (0.48–1.93)	0.80 (0.07–8.47)	0.94 (0.46–1.90)	1.07 (0.12–8.97)	0.74 (0.15–3.50)	0.96 (0.46–2.01)
<400^e^	1.00	1.00	1.00	1.00	1.00	1.00
≥400	0.91 (0.45–1.84)	1.25 (0.11–13.24)	0.89 (0.43–1.81)	1.50 (0.18–12.45)	0.91 (0.19–4.35)	0.91 (0.43–1.92)
**Dietary cobalamin**						
<2.4^e^	1.00	1.00	1.00	1.00	1.00	1.00
≥2.4	1.15 (0.51–2.59)	0.66 (0.42–1.05)	1.19 (0.51–2.73)	0.66 (0.44–0.99)	1.06 (0.15–7.14)	1.43 (0.60–3.36)
<3.7^d^	1.00	1.00	1.00	1.00	1.00	1.00
≥3.7	0.76 (0.37–1.52)	0.55 (0.31–0.99)	0.73 (0.35–1.48)	0.50 (0.28–0.88)	0.72 (0.15–3.38)	0.92 (0.43–1.94)
**Total cobalamin**						
<2.4^e^	1.00	1.00	1.00	1.00	1.00	1.00
≥2.4	0.97 (0.42–2.22)	0.66 (0.42–1.05)	0.98 (0.42–2.31)	0.66 (0.44–0.99)	0.75 (0.10–5.43)	1.29 (0.54–3.08)
<4.0^d^	1.00	1.00	1.00	1.00	1.00	1.00
≥4.0	0.71 (0.35–1.43)	**0.33 (0.13–0.84)**	0.68 (0.33–1.39)	**0.33 (0.15–0.74)**	0.87 (0.18–4.21)	1.00 (0.47–2.13)
**Residual intake (µg/d)** ^**f**^						
**Dietary folate**						
<374^f^	1.00	1.00	1.00	1.00	1.00	1.00
≥374	0.55 (0.27–1.10)	0.80 (0.07–8.47)	0.52 (0.25–1.06)	1.07 (0.12–8.97)	0.60 (0.12–2.83)	0.53 (0.25–1.13)
**Dietary cobalamin**						
<3.4^f^	1.00	1.00	1.00	1.00	1.00	1.00
≥3.4	0.86 (0.43–1.71)	0.44 (0.21–0.92)	0.77 (0.38–1.57)	**0.33 (0.15–0.74)**	1.09 (0.23–5.18)	1.05 (0.50–2.21)
**Total folate**						
<465^f^	1.00	1.00	1.00	1.00	1.00	1.00
≥465	0.91 (0.45–1.82)	0.80 (0.07–8.47)	0.88 (0.43–1.79)	1.07 (0.12–8.97)	0.74 (0.15–3.50)	0.90 (0.43–1.88)
**Total cobalamin**						
<3.8^f^	1.00	1.00	1.00	1.00	1.00	1.00
≥3.8	0.80 (0.40–1.60)	3.75 (0.27–51.37)	0.77 (0.38–1.57)	5.60 (0.47–66.44)	1.09 (0.23–5.18)	0.92 (0.43–1.94)

ER; estrogen receptor, PR; progesterone receptor, HER2; human epidermal growth factor receptor 2.

^a^The classification of angiopoietin 2 was based on median plasma level of our studied population.

^b^All variables were categorized in dichotomized as high versus low groups.

^c^Dietary and total intakes of folate were classified based on the cut of point obtained in our sample population.

^d^Dietary and total folate and cobalamin were classified based on the median intake of studied BC participants.

^e^Dietary and total folate and cobalamin intakes were classified based on dietary reference intake (DRI)^36^.

^f^Energy-adjusted models (residual). Just median-based classification of residual dietary and total folate and cobalamin intakes of BC studied population considered in this model. A statistically significant finding is shown in bold (*p-*value < 0.05).

### VEGF-C based categorization analyses

Plasma level of VEGF-C was categorized to dichotomous low and high levels (low: <0.06 and high: ≥0.06 ng/ml). Table 5 shows the correlation coefficients of dietary and total intakes of nutrients with plasma levels of Angiopoietins at either low or high VEGF-C sub-groups. Dietary and total intakes of folate indicated significantly to have inverse correlations with plasma levels of ANG-1 and ANG-2 in crude and adjusted models in the lower category of VEGF-C (Table 5). Similarly, dietary intake of cobalamin demonstrated an inverse correlation with ANG-2 in the crude model (β = −0.26, P < 0.05). Total cobalamin indicated inverse correlation in crude (β = −0.34, P < 0.05) and adjusted models (β = −0.33, P < 0.05). After including a group of patients with high VEGF-C, results showed that residual total cobalamin intake was positively associated with plasma ANG-2, after adjusting for related confounding factors (β = 0.31, P < 0.05; Table 5).

**Table 5:** The uni- and multivariate linear regression analyses were performed to obtain correlation coefficient between dietary, total and plasma level of folate and cobalamin intake in unadjusted and adjusted model with the plasma level of studied biomarkers and their ratio according to plasma level of VEGF-C (N = 177).

Variable	ANG2		ANG1		ANG1/Tie-2		ANG1/ANG2	
	Low VEGF-C ^a^	High VEGF-C	Low VEGF-C	High VEGF-C	Low VEGF-C	High VEGF-C	Low VEGF-C	High VEGF-C
Nutrient Intake (µg/d)								
Dietary folate								
Crude	**−** **0.31 (0.01)** ^**b**^	0.10 (0.40)^b^	**−0.30 (0.01)** ^**b**^	−0.01 (0.94)^b^	−0.03 (0.79)^b^	−0.08 (0.52)^b^	−0.02 (0.81)^b^	0.10 (0.40)
Model1^c^	**−0.29 (0.01** **)**	−0.13 (0.33)	**−0.35 (0.01)**	0.14 (0.29)	0.02 (0.98)	−0.07 (0.56)	0.12 (0.33)	0.17 (0.21)
Model2^d^	**−0.28 (0.04)**	−0.07 (0.66)	**−0.34 (0.01)**	0.19 (0.23)	−0.09 (0.49)	0.03 (0.81)	0.52 (0.09)	0.20 (0.19)
Total folate								
Crude	**−0.43 (0.01)**	−0.03 (0.80)	**−0.32 (0.01)**	0.01 (0.89)	**−0.23 (0.04)**	−0.10 (0.41)	−0.07 (0.53)	0.07 (0.52)
Model1	**−0.45 (0.01)**	−0.16 (0.21)	**−0.33 (0.01)**	0.10 (0.43)	−0.07 (0.54)	−0.11 (0.38)	−0.05 (0.68)	0.11 (0.38)
Model2	**−0.46 (0.01)**	−0.01 (0.92)	**−0.39 (0.01)**	0.04 (0.80)	−0.13 (0.38)	0.05 (0.74)	−0.01 (0.91)	0.16 (0.28)
DFE								
Crude	**−0.32 (0.01)**	−0.06 (0.60)	**−0.24 (0.01)**	0.02 (0.82)	**−0.24 (0.04)**	−0.04 (0.72)	−0.07 (0.53)	0.01 (0.98)
Model1	**−0.35 (0.01)**	−0.15 (0.24)	−0.23 (0.07)	0.05 (0.68)	−0.04 (0.72)	−0.01 (0.95)	−0.14 (0.28)	0.01 (0.98)
Model2	**−0.33 (0.03)**	−0.08 (0.61)	−0.30 (0.07)	−0.01 (0.94)	−0.03 (0.81)	0.01 (0.96)	−0.14 (0.36)	0.03 (0.79)
Dietary cobalamin								
Crude	**−0.26 (0.02)**	−0.13 (0.26)	0.01 (0.55)	−0.10 (0.42)	0.07 (0.53)	−0.02 (0.82)	−0.02 (0.82)	0.03 (0.78)
Model1	−0.25 (0.05)	−0.08 (0.52)	0.02 (0.83)	−0.15 (0.24)	0.09 (0.46)	0.07 (0.57)	−0.07 (0.59)	0.04 (0.71)
Model2	−0.22 (0.13)	0.05 (0.73)	0.11 (0.43)	−0.10 (0.49)	0.10 (0.46)	0.21 (0.13)	−0.08 (0.55)	−0.02 (0.78)
Total cobalamin								
Crude	**−0.34 (0.01)**	0.01 (0.90)	0.01 (0.95)	−0.20 (0.10)	−0.03 (0.76)	−0.07 (0.55)	−0.13 (0.26)	0.02 (0.86)
Model1	**−0.33 (0.01)**	0.06 (0.61)	0.02 (0.84)	−0.25 (0.05)	0.03 (0.78)	0.01 (0.94)	−0.24 (0.05)	0.02 (0.83)
Model2	−0.26 (0.06)	0.24 (0.13)	0.07 (0.64)	−0.26 (0.08)	0.02 (0.84)	0.09 (0.50)	−0.23 (0.11)	−0.02 (0.85)
Residual intake (µg/d)								
Dietary folate								
Crude	**−0.30 (0.01)**	0.06 (0.63)	**−0.31 (0.01)**	−0.07 (0.55)	0.01 (0.96)	−0.07 (0.53)	−0.05 (0.66)	0.07 (0.53)
Model1	**−0.30 (0.01)**	−0.21 (0.13)	**−0.39 (0.01)**	0.06 (0.64)	−0.01 (0.91)	−0.09 (0.51)	0.32 (0.12)	0.13 (0.34)
Model2	**−0.28 (0.04)**	−0.13 (0.44)	**−0.38 (0.01)**	0.08 (0.60)	−0.10 (0.45)	−0.02 (0.87)	0.10 (0.49)	0.17 (0.26)
Total folate								
Crude	**−0.36 (0.01)**	−0.04 (0.72)	**−0.28 (0.01)**	0.01 (0.99)	−0.22 (0.06)	−0.05 (0.64)	−0.08 (0.50)	0.02 (0.86)
Model1	**−0.38 (0.01)**	−0.18 (0.16)	**−0.29 (0.02)**	0.05 (0.66)	−0.04 (0.72)	−0.02 (0.83)	−0.10 (0.42)	0.03 (0.82)
Crude	**−0.28 (0.01)**	−0.16 (0.17)	−0.02 (0.82)	−0.18 (0.13)	0.04 (0.68)	−0.01 (0.96)	−0.01 (0.90)	0.01 (0.95)
Model1	**−0.28 (0.02)**	−0.13 (0.32)	−0.03 (0.79)	−0.22 (0.08)	0.01 (0.95)	0.04 (0.74)	−0.05 (0.67)	0.02 (0.86)
Model2	−0.23 (0.11)	−0.02 (0.89)	0.01 (0.98)	−0.18 (0.25)	0.05 (0.71)	0.12 (0.40)	−0.03 (0.80)	−0.12 (0.41)
Total cobalamin								
Crude	−0.22 (0.06)	0.13 (0.28)	0.02 (0.82)	**−0.24 (0.04)**	−0.02 (0.86)	−0.09 (0.43)	−0.22 (0.05)	0.01 (0.99)
Model1	−0.22 (0.07)	0.18 (0.17)	0.01 (0.95)	**−0.28 (0.03)**	−0.02 (0.83)	−0.08 (0.53)	**−0.34 (0.01)**	−0.09 (0.95)
Model2	−0.16 (0.24)	**0.31 (0.04)**	0.02 (0.87)	**−0.32 (0.03)**	−0.01 (0.92)	−0.08 (0.58)	**−0.34 (0.01)**	−0.05 (0.69)
Plasma level								
Folate (ng/ml)								
Crude	0.01 (0.93)	0.03 (0.74)	−0.16 (0.15)	−0.01 (0.87)	**−0.26 (0.02)**	−0.19 (0.11)	0.15 (0.16)	0.19 (0.11)
Model1	0.02 (0.85)	−0.01 (0.96)	−0.13 (0.28)	−0.06 (0.61)	−0.14 (0.24)	−0.20 (0.11)	**0.30 (0.01)**	0.18 (0.16)
Model2	−0.02 (0.86)	0.01 (0.91)	−0.20 (0.17)	−0.06 (0.67)	−0.11 (0.39)	−0.25 (0.08)	**0.35 (0.01)**	0.20 (0.15)
Cobalamin (pg/ml)								
Crude	0.01 (0.90)	−0.03 (0.76)	0.14 (0.21)	−0.01 (0.90)	−0.06 (0.56)	0.03 (0.80)	0.02 (0.83)	−0.04 (0.70)
Model1	0.01 (0.92)	0.02 (0.87)	0.20 (0.13)	−0.05 (0.67)	0.08 (0.52)	0.05 (0.67)	0.01 (0.90)	−0.02 (0.86)
Model2	−0.02 (0.89)	0.02 (0.88)	0.24 (0.15)	−0.14 (0.33)	0.12 (0.41)	0.16 (0.26)	0.01 (0.99)	0.01 (0.96)

ANG1; angiopoietin 1, ANG2; angiopoietin 2, VEGF-C; vascular endothelial growth factor-C, DFE; dietary folate equivalent

^a^Plasma VEGF-C was categorized based on the median plasma level of the studied population.

^b^Data were expressed as β (*p-value*).

^c^Adjusted for age at diagnosis (yr) and body mass index (BMI) at diagnosis (kg/m^2^).

^d^Adjusted for age at diagnosis (yr), body mass index (BMI) at diagnosis (kg/m^2^), frequency of live-birth delivery and grade of disease (І, ІІ, ІІІ).

Statistical significant finding is shown in **bold** (*p-*value < 0.05).

Dietary and total intake levels of folate demonstrated inverse correlations with plasma levels of ANG-1 in the lower category of VEGF-C (Table 5). Residual total cobalamin intake demonstrated significant inverse correlations with plasma levels of ANG-1 when plasma levels of VEGF-C were high. Among study participants with low VEGF-C, inverse correlations were observed between plasma folate and the ANG-1/Tie-2 ratio (β = −0.26, P < 0.05) and also between total folate intake and the ANG-1/Tie-2 ratio (β = −0.23, P < 0.05). Individuals within the low VEGF-C category, demonstrated an inverse association between residual total cobalamin intake and ANG-1/ANG-2 ratio, after adjusting for covariates (β = −0.34, P < 0.05). Plasma folate showed a significant positive correlation with ANG-1/ANG-2 ratio only in patients with low VEGF-C (β = 0.35, P < 0.05), after adjusting for related confounding factors (Table 5).

### Structural equation modeling

**Figure 4:**
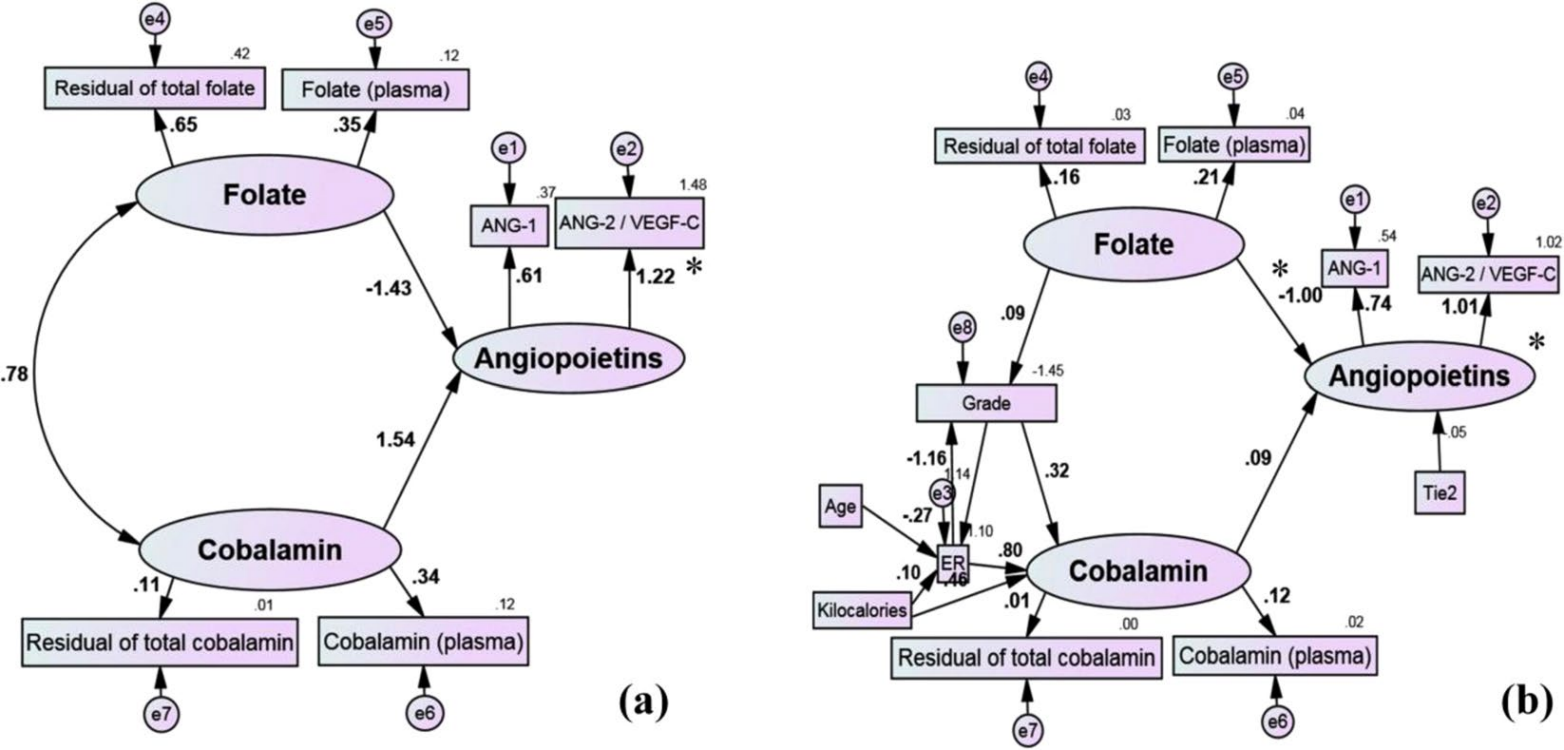
The proposed path diagram of the implied regression models fitted well in the parsimonious model (**a**) and just identified confirmatory multi-regression model (**b**) to show the contribution of folate and cobalamin with plasma Angiopoietins. Maximum likelihood estimation was performed to test the fitting function or estimation procedure of parameters in models. The plasma ANG-2 was weighted with interindividual levels of plasma VEGF-C. Rectangles were observed variables. Ellipses were latent (construct) variables. Values on the single-headed arrows (recursive) were standardized regression weights. Values on the double-headed arrows (nonrecursive) were beta (β) of intercorrelation between two variables. Each observed indicator included in models with measurement errors (**e**) and residual errors (top right corner of the rectangle) to predict the latent variable. Factor loading of a link between indicator and construct (latent) was also estimated. An asterisk (*) is shown to express critical ration (CR) >1.96 of estimated β, which means that the path (parameter) is significant at *p* < *0.05*.

The SEM was applied to determine a well-fitted model from the correlated variables. Primarily, a parsimonious model was identified showing the contributions of folate and cobalamin (as two exogenous latent variables) with Angiopoietins (an endogenous latent variable) (Fig. 4a). The model fitting indices were acceptable as CMIN/df <5, NFI ≤0.95, TLI = 1.00, CFI = 1.00 and RMSEA <0.05 (see Fig. 4). Each latent variable represented a construct of corresponding indicator variables which could be measured (measurement model) (Fig. 4a,b). The ANG-2 was intra-individually weighted for plasma VEGF-C (ANG-2/VEGF-C) to generate a transformed indicator. It was thus identified that the plasma level changes of Angiopoietins (latent) could be mediated inversely by the folate construct with a standard model weight (coefficient β) of −1.43 (P = 0.052, Fig. 4a). The independent cobalamin latent variable has a plausible positive correlation with the endogenous latent variable of Angiopoietins (β = 1.54, P = 0.054) (Fig. 4a). The level of significance was not acceptable indicating that these possible links may be influenced by unmeasured factors. Age at diagnosis and ER status were indirectly associated with plasma levels of folate and ANG-1 (Fig. 2b). Histological tumor grade was also considered a covariate factor. Plasma levels of folate featured prominently in the predicted measurement model of folate profile in the theoretical model (β = 0.370, P = 0.044; Fig. 4b). The ratio of ANG-2/VEGF-C featured significantly in the variance of Angiopoietins (latent variable) of β = 1.01 (P < 0.001; Fig. 4b). Confirmatory measurement model (multi-regression) resulted in a better goodness-of-fit to the data (CMIN/df <5,NFI <0.95, TLI = 0.724, CFI = 0.83 and RMSEA < 0.05; Fig. 2b). Adjustment for the indirect effects of age at diagnosis, average daily calorie intake, ER and histopathological grade of the tumor, the significant inverse contribution of folate profile (latent or construct variable) with plasma Angiopoietin changes (latent variable) (β = −0.999, P = 0.044) were observed.

### Effects of folic acid treatment on cell viability and transcription levels of ANGPTs, Tie-2 and VEGF genes in MCF-7 cells

**Figure 5:**
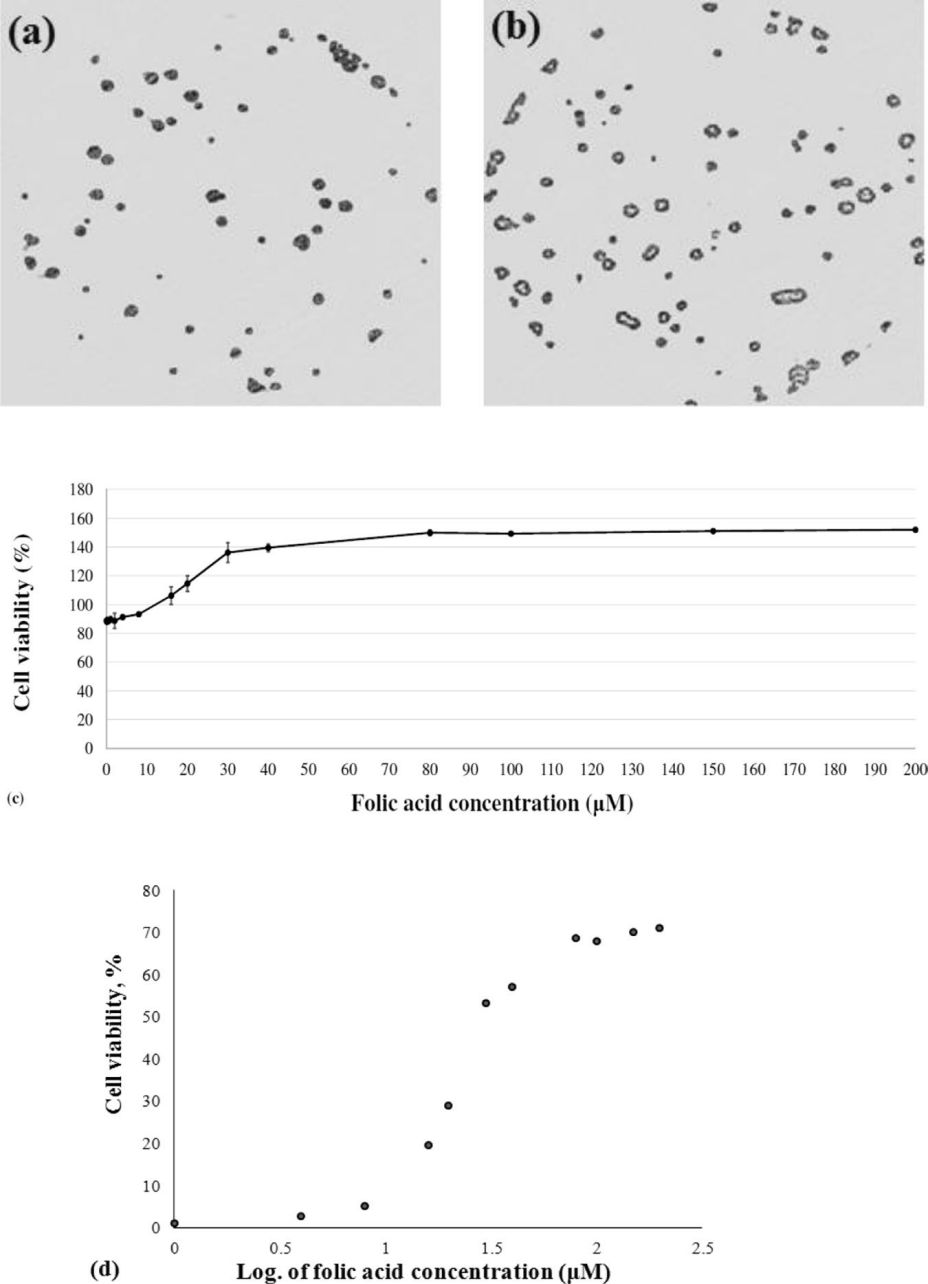
Microscope images (10 × ) of cologenic assays on MCF-7 cells at free folic acid medium as control (**a**) and folic acid concentration of 20 μM (**b**) after 5 days of treatments. Effects of folic acid treatment at different concentrations (0.1–200 μM) on the MCF-7 proportion of cell viability (**c**). The effects of different concentrations of folic acid were compared to control using ANOVA followed by Dunnet test (**c**). Calculated EC_50_ value to express the half-maximal viability of MCF-7 cells in response to folic acid treatment was 29.9 μM folic acid after 24 h. Data were expressed as the mean ± S.D. of three independent experiments.

Visualized images of clonogenic assays on MCF-7 cells at free folic acid medium (as control) and folic acid concentration at 20 μM after 5 days treatments were shown in Fig. 5(a,b). As a preliminary step, the effects of different concentrations of folic acid (0.1–200 μM) on cell viability of MCF-7 was examined after 24 h treatments (Fig. 5c). The percentage of cell numbers with active metabolism in experiments by using folic acid ≥16 μM was remarkably different from untreated control at P < 0.001 (Fig. 5c). The calculated EC_50_ value as a dose-response curve to express the half viability of MCF-7 cells (survival rate) in response to folic acid treatment existed at 29.9 μM concentration after 24 h (Fig. 5d).

**Figure 6:**
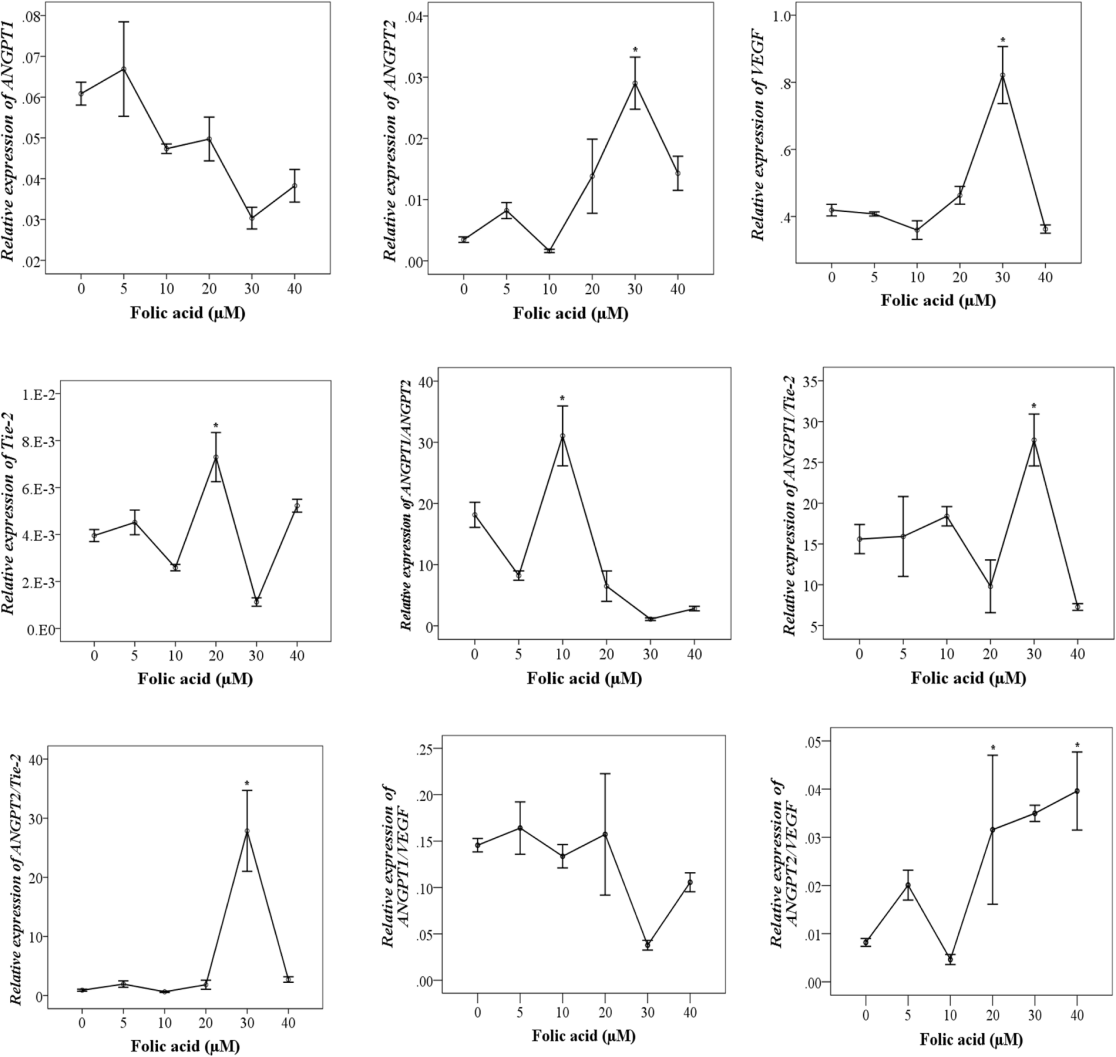
The effects of folic acid treatments in MCF-7 cells on expression levels of tested genes (*ANGPT1*, *ANGPT2*, *VEGF*, and *Tie-2*) relative to *HGPRT* as an internal control. Mean value of relative expression at each concentration of folic acid were represented at mean ± S.D. and compared using ANOVA followed by Dunnett analysis. (*p < *0.05* considered statistically significant).

Since folic acid treatment led to the growth of MCF-7 (Fig. 5c), we examined the effects of folic acid on the transcription levels of interested genes. Hence, Fig. 6 was plotted to illustrate trendlines showing changes in the relative expression levels of the tested gene (*ANGPT1*, *ANGPT2*, *VEGF and Tie-2*) dependent on ascending concentrations of folic acid treatments on MCF-7 cells. Folic acid-induced decreases in the relative expression levels of *ANGPT1* and increases in *ANGPT2* at 24 h after intervention in a concentration-dependent manner (Fig. 6). The ratio of *ANGPT1*/*ANGPT2* has also followed a descendant trend in response to rising folic acid concentrations (Fig. 6). The relative expression of the *VEGF* gene was also increased by increasing folic acid concentrations, and reached the summit particularly at 30 μM folic acid (vs. control, P < 0.05).

## Discussion

The present case-cohort series study has revealed new insights into the possible association of folate and cobalamin on plasma levels of tumor-derived regulating parameters which are important for lymphangiogenesis. The main finding of the present study demonstrates that dietary intake of folate is inversely associated with the plasma levels of both ANG-1 and ANG-2. In support of these findings, a theoretical confirmatory model was also developed defining folate profiles (the measurement model of plasma folate and residual total folate intake) as significant indicator contributed inversely to the Angiopoietins profile (the measurement model of plasma levels of ANG-1 and ANG-2/VEGF-C system).

The B vitamins (folate, and cobalamin) are well-known co-enzymes for thymidylate synthetase and purine synthesis^48^. Therefore, folate and cobalamin are in high demand for the rapid cellular proliferation required for ECs proliferation^29^. The folate-dependent proliferation of ECs has been focused by several studies with quite no back up describing the correlation of folate and Angiopoietins and VEGF in breast cancer cells^49,50^. This is the first study providing evidence that there is a significant inverse correlation between folate and plasma levels of Angiopoietins suggesting that the high folate intake may contribute to the presence of a high methyl density of regulatory DNA sequences and associated downregulation of tumoral Angiopoietins^34^. Although rapid tumor proliferation leads to overproduction of angiogenic factors^3,51^, there is compelling experimental *in vitro* evidence suggesting that the antiproliferative effects of folic acid treatment on human cell lines are mediated by the folate receptor (FR)/cSrc/ERK/NFκB/P53 pathway^28^. Activating the folate receptor pathway induces P21- and P27-dependent cell cycle arrest at the G1/S checkpoint^28^. This may contribute to the effects of folic acid on inhibiting tyrosine kinase activity (epidermal growth factor receptor) and related intracellular signaling in colon cancer cell lines^49^. In general, exposure to high folate is correlated with a reduced likelihood of raised plasma levels of ANG-1 and ANG-2 in breast cancer patients. Consistently, folic acid treatment in MCF-7 cells supported the decreasing trend of *ANGPT1* expression in a dose-dependent manner. By contrast, the increments in transcriptions of *ANGPT2* and *ANGPT2*/*Tie-2* axis induced by high concentrations of folic acid (particularly at >20 μM) was observed to support that extra physiologic concentration of folic acid might positively affect tumor-derived ANG-2-related lymphangiogenesis and lymphatic metastasis. However, folic acid (<10 μM) repressed expression of *ANGPT2*. The varied response rate of expressions seems to appear in a dose-dependent mode. In this regard, our findings showed that folic acid can induce downregulation in *ANGPT1* and *ANGPT2* levels *in vitro* when treatment was near physiologic doses (plasma: 2–20 nM/L,^52^) in favor of controlling angiogenesis. In agreement with present findings, a previous *in vitro* study^53^ demonstrated the potential dose-dependent inhibitory role of folic acid on downstream targets of PI3K/Akt signaling pathway including VEGF-A and IL-1β productions by which would actively be involved in pathologic hemoangiogenesis^53^. Indeed, under hypoxic condition, folic acid by interfering with the PI3K/Akt/HIF-1α pathway^53^ might decrease gene expression of proliferative and inflammatory factors such as CDK2 and possibly folic acid can take part potentially in protecting against tumor cell proliferation^53^. Moreover, the indirect inhibitory role of folate has been demonstrated on *VEGF-C* expression^54^. Indeed, folate is one of the primary precursors of S-adenosylmethionine (SAM) which is a predominant methyl donor component for numerous methylation reactions. An *in vitro* study has documented a reversing effects of SAM on *VEGF-C* gene and other oncogene promoters such as c-myc and H-ras hypomethylation which can effectively downregulate their expression and inhibit the growth of tumor cells^54^. Nevertheless, the optimum concentrations of folic acid to interfere breast tumor progression needs further experimental studies.

A study by Rykala *et al*.^19^ has demonstrated a significant correlation between the ER + subtype and the overregulation of *VEGF* among BC patients^19^. Indeed, ER affects tumoral progression via overexpressing proliferative factors^55^. The regulatory regions of *VEGFs* genes contain response elements for active ER by estrogenic metabolites and impress the recruitment of enhancers to overexpress proliferative growth factors^55^. In this regard, an *in vitro* study by Rickard *et al*.^56^ has reported significant up-regulatory effects of estrogen on expression levels of *VEGF-A* and *VEGF-C* through transcriptional mediating effects of ER signaling pathway^56^. Since ER is a prominent transcriptional factor enhances *PgR* transcription^57^, and thereby, PR could be assumed to have a contribution to induce the overexpression of growth factors dependent on ER activity. In contrast, Marton *et al*.^58^ indicated a significant negative association between PR positivity and *VEGF-C* expression^58^. However, it is worth mentioning that they provided results in very rare BC subtype, as neuroendocrine breast cancer (NEBC), presented that may cause inconsistency.

Accordingly, our findings showed that high levels of folate intake are associated with lower plasma levels of ANG-1 and ANG-2 in BC patients with a tumor hormonal receptor status of ER + /PR + . However, cobalamin intake indicated a significant inverse correlation with higher plasma levels of ANG-2 among BC patients diagnosed with ER-/PR- tumors. A confirmatory path analysis outlined that following consideration of the intercorrelation of age and ER status, folate profiles were inversely associated with Angiopoietins in breast cancer patients. Notably, the folate level can inversely associate with plasma levels of Angiopoietins, particularly among ER + patients who are prone to the VEGF-dependent elevated levels of angiopoietin. Consistently, *in vitro* treatments by folic acid in MCF-7 which is well-known as ER + /PR + breast adenocarcinoma cells, represented significant increased in *ANGPT2*/*VEGF* levels. Folic acid treatment at 10 μM, which is near the average plasma levels, was caused to a decline in the ratio of *ANGPT2*/*VEGF*. Therefore, interventions with high doses of folic acid seem to exert tumor progression dependent on upregulation of Angiopoietins and VEGF-C, whereas, low levels of folic acid can induce down-regulation in the *VEGF* gene and the ratio of *ANGPT2*/*VEGF*.

Notably, the elevated plasma levels of ANG-2 was higher than ANG-1 in the present study population. It is speculated that changes in ANG-1/ANG-2 ratio, in favor of increasing ANG-2, might associate with new vessel formation from pre-existing vasculature and is conditionally dependent on the presence of high levels of VEGFs^8,59,60^. Despite the predominant contribution of ANG-2 in inducing angiogenesis and lymphangiogenesis, ANG-1 is necessarily important for stabilizing the veins^1^. Both *in vitro* treatment effects and population-based findings were in agreement to show an inverse association between folate and ANG-1/*ANGPT1* levels in breast cancer and thereby may attenuate ECs integrity, migration, and maturation of newly formed blood vessels^8^. According to findings provided by Harfouche *et al*.^61^, estradiol (E2) that mediates the down-regulation of *ANGPT1* mRNA expression in breast cancer cell line could be responsible to present the lower degree of angiogenesis in ERα dependent specimens. In addition, high plasma levels of folate, unlike cobalamin, was correlated with high ANG-1/ANG-2 ratio implying that the preventive contribution of folate partly may have a tendency to act as a stabilizer of the vessel wall in order to sustain vessels^28^. Consistently, the rise in *ANGPT1*/*ANGPT2* ratio was only obtained at low concentrations of folic acid treatments *in vitro* (<10 μM). Though, high concentrations of folic acid even can act distinctly and decrease *ANGPT1*/*ANGPT2*, suggesting the effects of high folate might be important in promoting *ANGPT-2*-dependent pathologic angiogenesis.

Our findings showed that folate correlated inversely with the plasma levels of both ANG-1 and ANG-2 and ANG-1/ANG-2 ratio in the presence of low plasma level of VEGF-C. This supports our hypothesis independently of the growth-promoting effects of VEGF-C. However, Huang *et al*. indicated that when VEGF is low or being blocked, ANG-1 can stop the regression of tumor vessels^62^. Accordingly, our findings showed that high folate was associated with lower plasma levels of both Angiopoietins when VEGF-C levels were low. It is appealing to suggest that high folate may mask the potential growth effects of Angiopoietins by increasing the ANG-1/ANG-2 ratio in the circulation when VEGF-C is low. However, further research is warranted.

Residual total cobalamin showed an inverse correlation with ANG-1 levels among women with high plasma levels of VEGF-C. Cobalamin inversely could correlate with ANG-1/ANG-2 ratio in the presence of the downregulated VEGF-C, implying that higher intake of cobalamin might not protect pathological lymphangiogenesis in tumors^23,59^. Generally, peripheral and intratumoral lymphatic regression can occur when VEGF-C/VEGFR-3 is being inhibited^4^. More specifically, it was documented that repressing ANG-1 alone can not normalize blood vessel growth unless it occurs in combination with ANG-2 inhibition^23^. It seems that the presence of higher level of VEGF-C can distort the inverse correlation of folate and cobalamin with plasma levels of ANG-1 and ANG-2, suggesting that VEGF-C-related signal transduction can be crucial and a potent determinant in lymphangiogenesis.

Present findings showed that high plasma levels of folate correlate with a reduced ratio of ANG-1/Tie-2 only in patients with low levels of VEGF-C. This suggests that folate may be a contributing factor in reducing the availability of ANG-1 for ligand binding to Tie-2 and subsequent transduction of intracellular signaling for proliferation.

There were some limitations in our study. The results of subgroup analyses are interpreted cautiously since the subgroup samples are relatively small. Recall bias related to questionnaires is unavoidable in retrospective studies. To minimize some errors, we interviewed the patients before surgery (modified radical mastectomy) prior to a cancer diagnosis. Also, we provided the usual intake portions and dishes to assist participants to readily recall quantities and increase the accuracy of dietary data collection. Routine mandatory food fortification with folate or cobalamin was not instigated in Iran at the time of the study, so our intake estimations are independent of this covariate. Consumption of alcoholic beverages is prohibited among Iranian people because of cultural and religious rules. Therefore, we expect that results are less likely affected by the metabolic interactions of alcohol and folate fortification.

## Conclusions

In conclusion, this is the first study to evaluate the correlation of folate and cobalamin with ANGs, VEGF-C plasma levels and their interactions among BC patients. Plasma and dietary intake levels of folate indicated a significant inverse correlation with pro-angiogenic growth factors which are important for lymphangioenesis. However, cobalamin demonstrated results in favor of lower levels of ANG1/ANG2 ratio as a mediator of tumor progression. Generally, this observational epidemiologic study indicates a possible correlation of folate with low plasma levels of ANGs when blood VEGF-C is low. In contrast, high doses of folic acid *in vitro* (>20 μM) can multiply the transcription levels of *ANGPT2*/*Tie-2*, *VEGF-C*, and *ANGPT2*/*VEGF*-C in favor of showing lymphangiogenic effects of folic acid. Overall, results of the present study could support the previous evidence showing the preventive effects of folate on Angiopoietins/VEGF-dependent malignant breast cells propagation to develop metastasis.

## Supplementary information

Supplementary Table 1. The area under curve (AUC) for dietary and total folate and cobalamin intake (FFQ) obtained by using plasma levels of folate and cobalamin as biochemical indicators (n=177)

Supplementary Table 2. Nucleotide sequences of primers used in real-time PCR.

## Data Availability

The datasets used and/or analyzed during the current study are available from the corresponding author on reasonable request.

## References

[1] Fagiani E, Christofori G (2013). Angiopoietins in angiogenesis. Cancer Lett.

[2] Mitsuhashi N (2003). Angiopoietins and Tie‐2 expression in angiogenesis and proliferation of human hepatocellular carcinoma. Hepatology..

[3] Saharinen P, Eklund L, Pulkki K, Bono P, Alitalo K (2011). VEGF and angiopoietin signaling in tumor angiogenesis and metastasis. Trends Mol Med..

[4] Mattila MMT (2002). VEGF‐C induced lymphangiogenesis is associated with lymph node metastasis in orthotopic MCF‐7 tumors. Int. J. Cancer..

[5] Skobe M (2001). Induction of tumor lymphangiogenesis by VEGF-C promotes breast cancer metastasis. Nat Med..

[6] Schweiger T (2016). Increased lymphangiogenesis in lung metastases from colorectal cancer is associated with early lymph node recurrence and decreased overall survival. Clin Exp Metastasis..

[7] Szarvas T (2009). Serum levels of angiogenic factors and their prognostic relevance in bladder cancer. Pathol Oncol Res..

[8] Staton CA (2011). Angiopoietins 1 and 2 and Tie‐2 receptor expression in human ductal breast disease. Histopathol..

[9] Meher A, Sundrani D, Joshi S (2015). Maternal nutrition influences angiogenesis in the placenta through peroxisome proliferator activated receptors: a novel hypothesis. Mol Reprod Dev..

[10] Varney ML, Singh RK (2015). VEGF-C-VEGFR3/Flt4 axis regulates mammary tumor growth and metastasis in an autocrine manner. Am J Cancer Res..

[11] Simiantonaki N (2008). Hypoxia-induced epithelial VEGF-C/VEGFR-3 upregulation in carcinoma cell lines. Int. J. Oncol..

[12] Zhang J (2016). Macrophage migration inhibitory factor regulating the expression of VEGF-C through MAPK signal pathways in breast cancer MCF-7 cell. World J SURG ONCOL..

[13] Ni X (2013). Hypoxia-induced factor-1 alpha upregulates vascular endothelial growth factor C to promote lymphangiogenesis and angiogenesis in breast cancer patients. J Biomed Res..

[14] Carmeliet P, Jain RK (2011). Molecular mechanisms and clinical applications of angiogenesis. Nature medicine..

[15] Albuquerque RJ (2013). The newest member of the VEGF family. Blood..

[16] Koch S, Tugues S, Li X, Gualandi L, Claesson-Welsh L (2011). Signal transduction by vascular endothelial growth factor receptors. Biochem. J..

[17] Thurston G (2003). Role of angiopoietins and Tie receptor tyrosine kinases in angiogenesis and lymphangiogenesis. Cell Tissue Res..

[18] Helfrich I (2009). Angiopoietin-2 levels are associated with disease progression in metastatic malignant melanoma. Clin Cancer Res..

[19] Rykala J (2011). Angiogenesis markers quantification in breast cancer and their correlation with clinicopathological prognostic variables. Pathol Oncol Res..

[20] Zheng W (2014). Angiopoietin 2 regulates the transformation and integrity of lymphatic endothelial cell junctions. Genes Dev..

[21] Dellinger M (2008). Defective remodeling and maturation of the lymphatic vasculature in Angiopoietin-2 deficient mice. Dev. Biol..

[22] Chen H-M, Tsai C-H, Hung W-C (2015). Foretinib inhibits angiogenesis, lymphangiogenesis and tumor growth of pancreatic cancer *in vivo* by decreasing VEGFR-2/3 and TIE-2 signaling. Oncotarget..

[23] Saharinen P, Bry M, Alitalo K (2010). How do angiopoietins Tie in with vascular endothelial growth factors?. Curr Opin Hematol..

[24] Alawo DO (2017). Regulation of angiopoietin signalling by soluble Tie2 ectodomain and engineered ligand trap. Sci Rep..

[25] Cheng F (2016). Folic acid attenuates vascular endothelial cell injury caused by hypoxia via the inhibition of ERK1/2/NOX4/ROS pathway. Cell Biochem. Biophys..

[26] Mahan, L. K., Raymond, J. L. Krause’s food & the nutrition care process. (Elsevier Health Sciences, 2016).

[27] Schindler R, Mentlein R (2006). Flavonoids and vitamin E reduce the release of the angiogenic peptide vascular endothelial growth factor from human tumor cells. J Nutr..

[28] Lin SY (2012). Folic acid inhibits endothelial cell proliferation through activating the cSrc/ERK 2/NF-κB/p53 pathway mediated by folic acid receptor. Angiogenesis..

[29] Pirouzpanah S, Taleban FA, Abadi AR, Atri M, Mehdipour P (2009). The association of plasma folate, vitamin B12 and homocysteine levels on hypermethylation status of rarβ2 gene in primary breast carcinoma. Iran J Epidemiol..

[30] Mahmoud AM, Ali MM (2019). Methyl Donor Micronutrients that Modify DNA Methylation and Cancer Outcome. Nutrients..

[31] Zhang CX (2011). Dietary folate, vitamin B 6, vitamin B 12 and methionine intake and the risk of breast cancer by oestrogen and progesterone receptor status. Br J Nutr..

[32] Pirouzpanah S, Taleban FA, Atri M, Abadi AR, Mehdipour P (2010). The effect of modifiable potentials on hypermethylation status of retinoic acid receptor-beta2 and estrogen receptor-alpha genes in primary breast cancer. Cancer Causes Control..

[33] Montazeri V (2016). Reproductive risk factors of breast cancer among women in Tehran and Northwest of Iran: a case-control study. Iran J Epidemiol..

[34] Pirouzpanah S, Taleban FA, Mehdipour P, Atri M (2015). Association of folate and other one-carbon related nutrients with hypermethylation status and expression of RARB, BRCA1, and RASSF1A genes in breast cancer patients. J Mol Med..

[35] Pirouzpanah S, Taleban FA, Mehdipour P, Atri M, Foroutan-Ghaznavi M (2014). Plasma total homocysteine level in association with folate, pyridoxine, and cobalamin status among Iranian primary breast cancer patients. Nutr cancer..

[36] Peracchi M, Bamonti Catena F, Pomati M, De Franceschi M, Scalabrino G (2001). Human cobalamin deficiency: alterations in serum tumour necrosis factor‐α and epidermal growth factor. Eur J Haematol..

[37] Committee, N. E. A. *Ethical Guidelines for Observational Studies. Observational research, audits and related activities. Revised edition. July 2012*. 2014.

[38] Von Elm E (2007). The Strengthening the Reporting of Observational Studies in Epidemiology (STROBE) statement: guidelines for reporting observational studies. PLoS medicine..

[39] Williams LA (2019). Differences in race, molecular and tumor characteristics among women diagnosed with invasive ductal and lobular breast carcinomas. Cancer Causes Control..

[40] Bauer KR, Brown M, Cress RD, Parise CA, Caggiano V (2007). Descriptive analysis of estrogen receptor (ER)‐negative, progesterone receptor (PR)‐negative, and HER2‐negative invasive breast cancer, the so‐called triple‐negative phenotype: a population‐based study from the California cancer Registry. Cancer..

[41] Parise Carol A., Caggiano Vincent (2014). Breast Cancer Survival Defined by the ER/PR/HER2 Subtypes and a Surrogate Classification according to Tumor Grade and Immunohistochemical Biomarkers. Journal of Cancer Epidemiology.

[42] Pirouzpanah S (2014). The biomarker-based validity of a food frequency questionnaire to assess the intake status of folate, pyridoxine and cobalamin among Iranian primary breast cancer patients. Eur J Clin Nutr..

[43] Willett WC, Stampfer MJ (1986). Total energy intake: implications for epidemiologic analyses. Am J Epidemiol..

[44] Schmittgen TD, Livak KJ (2008). Analyzing real-time PCR data by the comparative C(T) method. Nat Protoc.

[45] Bentler PM, Bonett DG (1980). Significance tests and goodness of fit in the analysis of covariance structures. Psychol Bull..

[46] Marsh HW, Balla JR, McDonald RP (1988). Goodness-of-fit indexes in confirmatory factor analysis: the effect of sample size. Psychol Bull..

[47] Jung K (2006). Tietz Textbook of Clinical Chemistry and Molecular Diagnostics. MO: Elsevier Saunders, 2006, 2448 pp., $229.00, hardcover. ISBN 0-7216-0189-8. Clin Chem..

[48] Li B, Lu Y, Wang L, Zhang CX (2015). Folate intake and breast cancer prognosis: a meta-analysis of prospective observational studies. EUR J Cancer Prev..

[49] Jaszewski R (1999). Folic acid inhibition of EGFR-mediated proliferation in human colon cancer cell lines. AM J Physiol Cell Physiol..

[50] Kuo, C. T., Chang, C., Lee, W. S. Folic acid inhibits COLO-205 colon cancer cell proliferation through activating the FRα/c-SRC/ERK1/2/NFκB/TP53 pathway: *in vitro* and *in vivo* studies. *Sci Rep* **5** (2015). 10.1038/srep11187 PMC4460902 26056802

[51] Stockmann C (2008). Deletion of vascular endothelial growth factor in myeloid cells accelerates tumorigenesis. Nature..

[52] Fishback, F., Dunning, M. Manual of laboratory and diagnostic test. *Milwaukee: Lippincott*. **636** (1992).

[53] Huang X (2016). Folic acid represses hypoxia-induced inflammation in THP-1 cells through inhibition of the PI3K/Akt/HIF-1α pathway. PloS one..

[54] Da M, Zhang Y, Yao J, Duan Y (2014). DNA methylation regulates expression of VEGF-C, and S-adenosylmethionine is effective for VEGF-C methylation and for inhibiting cancer growth. Braz J Med Biol Res..

[55] Khaja ASS (2013). Cyclin A1 modulates the expression of vascular endothelial growth factor and promotes hormone-dependent growth and angiogenesis of breast cancer. PloS One..

[56] Rickard DJ (2002). Estrogen receptor isoform‐specific induction of progesterone receptors in human osteoblasts. J Bone Miner Res..

[57] Pirouzpanah, S., Taleban, F.-A., Mehdipour, P., Sabour, S., Atri, M. Hypermethylation pattern of ESR and PgR genes and lacking estrogen and progesterone receptors in human breast cancer tumors: ER/PR subtypes. *Cancer Biomark*. 1–18 (2018). 10.3233/CBM-170697 PMC13078300 29278880

[58] Marton I, Knežević F, Ramić S, Milošević M, Tomas D (2012). Immunohistochemical expression and prognostic significance of HIF-1α and VEGF-C in neuroendocrine breast cancer. Anticancer Res..

[59] Hashizume H (2010). Complementary actions of inhibitors of angiopoietin-2 and VEGF on tumor angiogenesis and growth. Cancer Res.

[60] Tsutsui S (2006). Angiopoietin 2 expression in invasive ductal carcinoma of the breast: its relationship to the VEGF expression and microvessel density. Breast Cancer Res Treat..

[61] Harfouche R (2011). Estradiol-dependent regulation of angiopoietin expression in breast cancer cells. J Steroid Biochem Mol Biol..

[62] Huang J (2009). Angiopoietin-I/Tie-2 activation contributes to vascular survival and tumor growth during VEGF blockade. Int J Oncol..

