# Supplementary material for: The contribution of dietary and plasma folate and cobalamin to levels of angiopoietin-1, angiopoietin-2 and Tie-2 receptors depend on vascular endothelial growth factor status of primary breast cancer patients
Source: Sci Rep. 2019 Oct 16;9:14851. doi: 10.1038/s41598-019-51050-x (PMC6795805; doi:10.1038/s41598-019-51050-x)
Supplement: Supplementary file 1 — Supplementary Table 1. The area under curve (AUC) for dietary and total folate and cobalamin intake (FFQ) obtained by using plasma levels of folate and cobalamin as biochemical indicators (n=177) [file 41598_2019_51050_MOESM1_ESM.pdf]

The contribution of dietary and plasma folate and cobalamin to levels of angiopoietin-1, angiopoietin-2 and Tie-2 receptors depend on vascular endothelial growth factor status of primary breast cancer patients

Saeed Pirouzpanah, Parisa Varshosaz, Ashraf Fakhrjou, Vahid Montazeri

*** Corresponding Author:**

Saeed Pirouzpanah, M.Sc., Ph.D. Drug Applied Research Center/ and also Department of Biochemistry and Dietetics, Faculty of Nutrition and Food Sciences, Tabriz University of Medical Sciences, Tabriz 5166614711, Iran.

Tel: +9841-33357580-3

Fax: +9841-33340634

Email: pirouzpanah@gmail.com, and pirouzpanahs@tbzmed.ac.ir

Supplementary Table 1. The area under the curve (AUC) for dietary and total folate and cobalamin intakes (FFQ) obtained by using plasma levels of folate and cobalamin as biochemical indicators (n=177).

Plasma concentration/ nutrient intake	AUC	SE	*P-value*	95%CI	COP (μg/d)
**Folate (ng/ml)**					
**4.5 (reference)**					
Dietary folate	0.67	0.07	0.015	0.53-0.81	280
Total folate	0.71	0.06	0.003	0.58-0.84	280
DFE	0.71	0.06	0.003	0.58-0.84	257
Res. dietary folate	0.66	0.07	0.019	0.52-0.81	374
Res. total folate	0.71	0.06	0.003	0.58-0.85	465
**10.0 (median)**					
Dietary folate	0.66	0.04	<0.001	0.57-0.74	280
Total folate	0.64	0.04	0.001	0.56-0.73	280
Res. dietary folate	0.65	0.04	<0.001	0.57-0.74	331
Res. total folate	0.64	0.04	0.001	0.56-0.73	374
**Cobalamin (pg/ml)**					
**200 (reference)**					
Dietary cobalamin	0.58	0.05	0.097	0.47-0.69	2.63
Total cobalamin	0.56	0.05	0.227	0.45-0.67	2.08
Res. dietary cobalamin	0.57	0.05	0.146	0.46-0.68	3.34
Res. total cobalamin	0.55	0.05	0.268	0.45-0.66	3.84
**256 (median)**					
Dietary cobalamin	0.56	0.04	0.128	0.48-0.65	2.65
Total cobalamin	0.57	0.04	0.104	0.48-0.65	2.65
Res. dietary cobalamin	0.55	0.04	0.250	0.46-0.63	3.31
Res. total cobalamin	0.56	0.04	0.157	0.47-0.65	3.83

Abbreviations: COP; cut off point, n; number of patients, *Res.*; residual.
Plasma concentration of folate ≤ 4.5 ng/ml, cobalamin ≤ 200 pg/ml, were defined as deficiencies reference values [folate 2-20 ng/ml and cobalamin 200-900 pg/ml according to the protocol of kits (folate: Cat N. 7525-300 and cobalamin: Cat N. 7526-300)].
