# Supplementary material for: The contribution of dietary and plasma folate and cobalamin to levels of angiopoietin-1, angiopoietin-2 and Tie-2 receptors depend on vascular endothelial growth factor status of primary breast cancer patients
Source: Sci Rep. 2019 Oct 16;9:14851. doi: 10.1038/s41598-019-51050-x (PMC6795805; doi:10.1038/s41598-019-51050-x)
Supplement: Supplementary file 2 — Supplementary Table 2. Nucleotide sequences of primers used in real-time PCR. [file 41598_2019_51050_MOESM2_ESM.pdf]

The contribution of dietary and plasma folate and cobalamin to levels of angiopoietin-1, angiopoietin-2 and Tie-2 receptors depend on vascular endothelial growth factor status of primary breast cancer patients

Saeed Pirouzpanah, Parisa Varshosaz, Ashraf Fakhrjou, Vahid Montazeri

*** Corresponding Author:**

Saeed Pirouzpanah, M.Sc., Ph.D. Drug Applied Research Center/ and also Department of Biochemistry and Dietetics, Faculty of Nutrition and Food Sciences, Tabriz University of Medical Sciences, Tabriz 5166614711, Iran.

Tel: +9841-33357580-3

Fax: +9841-33340634

Email: pirouzpanah@gmail.com, and pirouzpanahs@tbzmed.ac.ir

Supplementary Table 2. Nucleotide sequences of primers used in the real-time PCR.

Gene	Forward primer	Reverse primer	Product size (bp)	Accession number
ANGPT1	5'-TTAACAGGAGGATGGTGGTTT-3'	5'-GGTTTTGTCCCGCAGTATAGA-3'	76	NM_001146.4(2)
ANGPT2	5'-ATGTCCACATCAAACTCTAA-3'	5'-ATTTGAATACTTCAGCACAG-3'	82	NM_001118887.1
Tie-2	5'-AAGTACACCTGCCTCATGCTCA-3'	5'-GGCTTCACATCTCCGGACTATC-3'	116	NM_000459.4
VEGF	5'-CTACCTCCACCATGCCAAGT-3'	5'-CCACTTCGTGATGATTCTGC-3'	74	NM_001025366.2
HGPRT	5'-TGGACAGGACTGAACGTCTTG-3'	5'- CCAGCAGGTCAGCAAAGAATTTA-3'	111	NM_000194(3)

ANGPT1, *angiopoietin-1*; *ANGPT2*, *angiopoietin-2*; *Tie-2*, *TEK tyrosine kinase-2*; *VEGF*, *vascular endothelial growth factor*; *HGPRT*, *hypoxanthine-guanine phosphoribosyltransferase*; bp, base pair.
